# *Atrx* deletion impairs CGAS/STING signaling and increases sarcoma response to radiation and oncolytic herpesvirus

**DOI:** 10.1172/JCI149310

**Published:** 2023-07-03

**Authors:** Warren Floyd, Matthew Pierpoint, Chang Su, Rutulkumar Patel, Lixia Luo, Katherine Deland, Amy J. Wisdom, Daniel Zhu, Yan Ma, Suzanne Bartholf DeWitt, Nerissa T. Williams, Alexander L. Lazarides, Jason A. Somarelli, David L. Corcoran, William C. Eward, Diana M. Cardona, David G. Kirsch

**Affiliations:** 1 Department of Pharmacology and Cancer Biology, and; 2 Department of Radiation Oncology, Duke University Medical Center, Durham, North Carolina, USA.; 3 Department of Radiation Oncology, Brigham and Women’s Hospital/Dana-Farber Cancer Institute, Boston, Massachusetts, USA.; 4 Department of Orthopaedics and; 5 Department of Medicine, Duke University Medical Center, Durham, North Carolina, USA.; 6 Department of Sarcoma, Moffitt Cancer Center, Tampa, Florida, USA.; 7 Duke Cancer Institute, Durham, North Carolina, USA.; 8 Center for Genomic and Computational Biology, Duke University, Durham, North Carolina, USA.; 9 Department of Pathology, Duke University Medical Center, Durham, North Carolina, USA.; 10 Radiation Medicine Program, Princess Margaret Cancer Centre, University Health Network, Toronto, Ontario, Canada.; 11 Department of Radiation Oncology and; 12 Department of Medical Biophysics, University of Toronto, Toronto, Ontario, Canada.

**Keywords:** Oncology, Innate immunity, Mouse models, Radiation therapy

## Abstract

*ATRX* is one of the most frequently altered genes in solid tumors, and mutation is especially frequent in soft tissue sarcomas. However, the role of *ATRX* in tumor development and response to cancer therapies remains poorly understood. Here, we developed a primary mouse model of soft tissue sarcoma and showed that *Atrx-*deleted tumors were more sensitive to radiation therapy and to oncolytic herpesvirus. In the absence of *Atrx*, irradiated sarcomas had increased persistent DNA damage, telomere dysfunction, and mitotic catastrophe. Our work also showed that *Atrx* deletion resulted in downregulation of the CGAS/STING signaling pathway at multiple points in the pathway and was not driven by mutations or transcriptional downregulation of the CGAS/STING pathway components. We found that both human and mouse models of *Atrx-*deleted sarcoma had a reduced adaptive immune response, markedly impaired CGAS/STING signaling, and increased sensitivity to TVEC, an oncolytic herpesvirus that is currently FDA approved for the treatment of aggressive melanomas. Translation of these results to patients with *ATRX-*mutant cancers could enable genomically guided cancer therapy approaches to improve patient outcomes.

## Graphical abstract

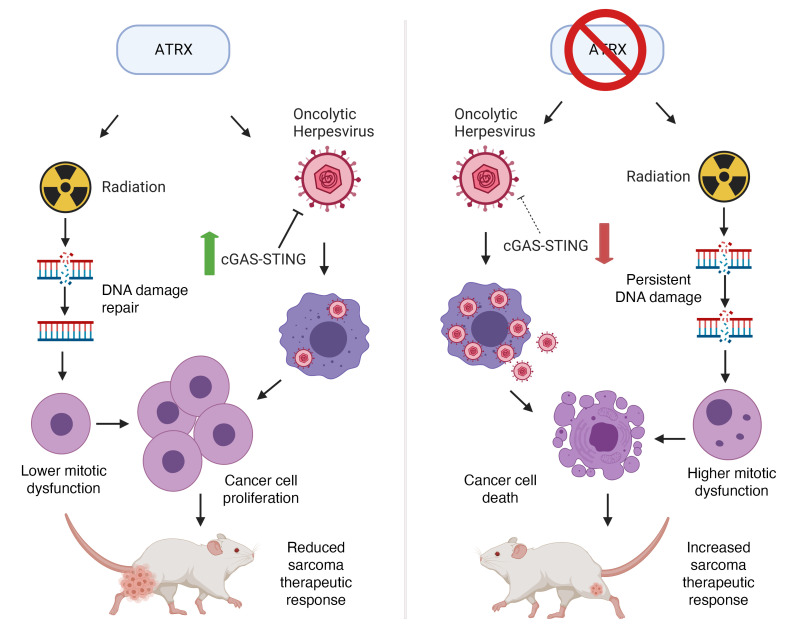

## Introduction

ATRX, or α-thalassemia/mental retardation, X-linked, is a chromatin remodeling protein and tumor suppressor, with mutations or copy number alterations occurring in approximately 6% of the more than 65,163 tumors sequenced in the AACR GENIE database (1). Mutations in *ATRX* are predominantly loss of function and are enriched in specific cancer types, including low-grade gliomas, soft tissue sarcomas, pancreatic neuroendocrine tumors, and uterine corpus endometrial carcinomas (2–5). The effect of ATRX on overall survival (OS) varies by cancer type. *ATRX* mutation is associated with improved OS in gliomas and worse OS in pancreatic neuroendocrine tumors (6, 7).

ATRX is hypothesized to play key roles in both normal and cancer cells, such as global epigenetic maintenance of pericentromeric heterochromatin, repression of alternative lengthening of telomeres (ALT), targeting of polycomb repressor complex 2, and regulation of DNA double strand break (DSB) damage repair (8–10). Recent studies have identified multiple methods by which ATRX regulates DNA damage repair. Recently, ATRX mediated histone 3.3 (H3.3) chromatin deposition was demonstrated to be an important regulator of homologous recombination (HR) and sister chromatid exchanges to exogenous double strand breaks in HeLa cells (11). ATRX mediates this effect via interactions with PCNA and RFC-1, which are collectively required for HR DNA repair. Additional studies have identified ATRX as a regulator of replication fork processivity and have further shown that *ATRX* knockdown results in impaired localization of RAD51 to BRCA, a key requirement for HR (12, 13). There are conflicting reports regarding the impact of *Atrx* deletion on radiation response, with increased radiosensitivity reported in some, but not all, studies (10, 14). Further work has demonstrated that ATRX plays an important role in maintaining telomere genomic stability in embryonic stem cells and in neuroprogenitor cells (10).

Interestingly, recent reports have identified ATRX as an important modulator of the cyclic guanosine monophosphate-adenosine monophosphate synthase (CGAS) and its adaptor protein Stimulator of IFN Gene (STING) pathway’s response to extrachromosomal telomeric repeat DNA in cancer cell lines with alternative lengthening of telomeres (ALT) (15). The CGAS/STING pathway acts as an innate immune sensor for microbial and viral pathogens by detecting dsDNA in the cytoplasm of mammalian cells and has emerged as an important link between DNA damage and the innate immune system (16–18). Engagement of the CGAS/STING pathway results in the phosphorylation of IFN regulatory factor 3 (IRF3) and transcriptional induction of type-I IFNs (19).

To study the role of ATRX in cancer development and therapeutic response, we generated a primary genetically engineered mouse model of soft tissue sarcoma with or without *Atrx* deletion in tumor cells with deletion of both alleles of *Trp53* and activation of oncogenic *Kras^G12D^*. We found that *Atrx* deletion increased radiation-induced persistent DNA damage, mitotic dysfunction, and radiotherapy response in both immune proficient and T cell deficient mouse models. We further showed that *Atrx-*deleted primary sarcomas have impaired adaptive immune response and reduced tumor-intrinsic CGAS/STING signaling after radiation. Finally, we demonstrated that sarcomas with *Atrx* deletion and aberrant CGAS/STING signaling were sensitized to oncolytic herpesvirus therapy. Taken together, these findings have what we believe to be important implications for precision oncology in *ATRX-*mutant cancers.

## Results

**Figure 1:**
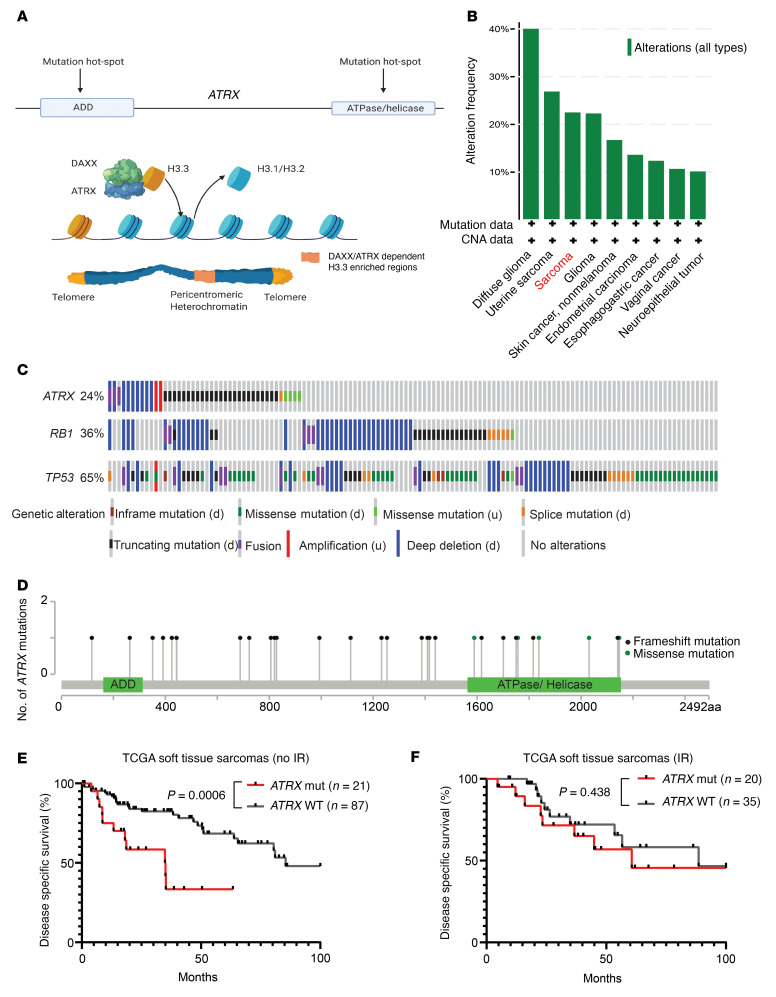
Genomic characteristics of *ATRX* in human cancer. (**A**) Diagram with 2 major functional domains of ATRX (ADD and ATPase/helicase). ATRX complexes with DAXX to promote deposition of histone 3.3 throughout the genome, with especially high concentrations at constitutive heterochromatin. (**B**) Alterations in *ATRX* in human cancers from TCGA sequencing database. (**C**) Schematic of the most frequently mutated genes in a human soft tissue sarcoma cohort comprising leiomyosarcoma, myxofibrosarcoma, and undifferentiated pleomorphic sarcoma (STS cohort), in TCGA sequencing database with each vertical row representing a single tumor sample. (d) denotes this class of mutations as a putative driver mutation, while (u) denotes this class of mutations as being of unknown functional impact. (**D**) Positional distribution of mutations in the *ATRX* gene from the STS cohort from TCGA. Black lollipop markers represent insertion or deletion mutations, while green lollipop markers represent missense mutations. (**E**) Disease-specific survival for an unirradiated STS cohort, comparing tumors with *ATRX* genomic alterations (*n* = 21) and tumors with WT ATRX (*n* = 87). (**F**) Disease-specific survival for a STS cohort in which tumors received radiation therapy, comparing tumors with *ATRX* genomic alterations (*n* = 20) and tumors with WT *ATRX* (*n* = 35). All statistical comparisons for this figure were performed using a log-rank (Mantel-Cox) test.

To model *ATRX* alterations from human cancer in a primary mouse model, we examined the Cancer Genome Atlas (TCGA), a large next-generation sequencing cancer database (4). Based on TCGA, we determined that 3 of the most common subtypes of soft tissue sarcoma, undifferentiated pleomorphic sarcoma, myofibrosarcoma, and leiomyosarcoma (STS cohort), had recurrent alterations in the *ATRX* gene, which occurred in 24% of samples. The majority of these *ATRX* alterations were copy number deletions, frameshift mutations, or missense mutations located within the gene’s functional domains (Figure 1, A–D) (5). We, therefore, concluded that *ATRX* alterations in human cancer can be faithfully modeled via conditional deletion, and that soft tissue sarcoma is a relevant model system in which to study the impact of *ATRX* loss-of-function mutations in cancer. Next, we found that, within this STS cohort in TCGA, *ATRX* alteration was associated with significantly worse disease–specific survival (Supplemental Figure 1A; supplemental material available online with this article; https://doi.org/10.1172/JCI149310DS1). This worsened disease-specific survival associated with ATRX alteration was even more pronounced in tumors that did not receive ionizing radiation (Figure 1E). Interestingly, however, survival was similar for patients with sarcomas with and without *ATRX* alterations when all patients were treated with radiation therapy (Figure 1F), suggesting that a *ATRX* loss-of-function mutation may increase the radiation response of soft tissue sarcomas. Mutations of genes such as *TP53*, *KRAS*, and *RB* that frequently cooccur with *ATRX* mutation did not result in a similar increase in radiation response, further suggesting that *ATRX* loss-of-function mutations are truly associated with an altered radiation response in human soft tissue sarcomas. Furthermore, we examined a publicly available data set of whole genome sequencing from human undifferentiated pleomorphic sarcoma (UPS), one of the most commonly diagnosed subtypes of soft tissue sarcoma in adults (2). Analysis of this data set revealed that human UPS with *ATRX* alterations had a significant increase in chromosomal rearrangements and an increased likelihood of SBS3, a mutational signature that reflects a defect in DNA double strand break (DSB) repair (Supplemental Figure 1, B and C) (20). In conjunction with previously published work (9, 11, 21, 22), these findings motivated us to generate a mouse model of *Atrx-*deleted sarcoma and examine the response to DNA DSB–inducing therapeutics.

### Generation and characterization of a primary mouse model of soft tissue sarcoma with Atrx deletion.

**Figure 2:**
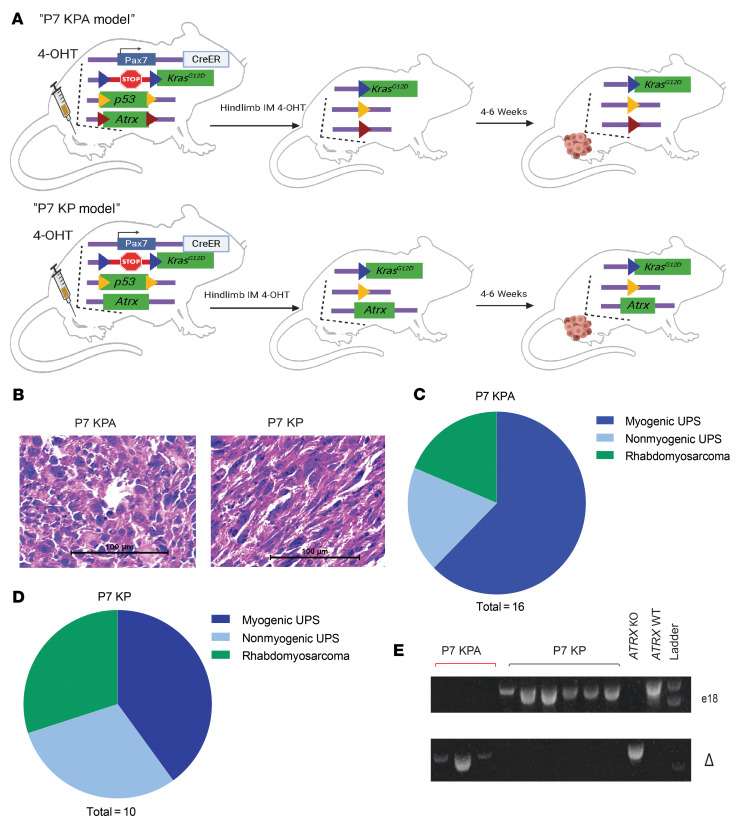
Primary mouse model of Atrx-deleted soft tissue sarcoma. (**A**) Schematic showing the spatially and temporally restricted primary mouse model of *Atrx*-deleted soft tissue sarcoma. 4-hydroxytamoxifen (4-OHT) is injected into the gastrocnemius muscle, which leads to activation of the CreER expressed from the endogenous *Pax7* promoter in muscle satellite cells to activate expression of *Kras^G12D^* and delete floxed *Trp53* and *Atrx* alleles. *Atrx* is deleted in P7 KPA mice (top) and a WT *Atrx* is retained in control P7 KP mice (bottom). Loxp sites are designated by colored triangles in the diagram. (**B**) H&E staining of sarcomas from P7 KPA and P7 KP mice. These H&E images are also shown in Supplemental Figure 2A (left) accompanied by staining for myogenic markers. (**C** and **D**) Classification of tumor type for P7 KPA (*n* = 16) and P7 KP (*n* = 10) tumors, as determined by IHC for 9 myogenic and other markers. Myogenic UPS was defined as when cells had pleomorphic nuclei characteristic of UPS but stained positive for at least 2 of the 4 tested myogenic markers (MyoD1, Myogenin, Desmin, and SMA). (**E**) Genotyping assays to confirm complete deletion of *Atrx* in the P7 KPA tumor model. Genotyping of sarcoma cell lines from P7 KP and P7 KPA mouse tumors for the presence of loxP flanked *Atrx* exon 18. The top genotyping gel portion shows the presence or absence of the portion of the *Atrx* band targeted for excision by the Cre/loxP system, with absence of a band in E1 indicating successful deletion of exon 18 of *Atrx*. To confirm the findings in the top gel, an additional genotyping assay (bottom) was performed that selectively amplified only the sequence that occurs after successful deletion of exon 18. The presence of a band in the bottom gel indicates successful deletion of *Atrx* exon 18.

To study the effect of *Atrx* in soft tissue sarcoma, we adapted a spatially and temporally restricted primary mouse model of soft tissue sarcoma (P7 KPA model) (23). This model utilizes an estrogen receptor-regulated Cre-recombinase that is expressed from the muscle satellite cell-specific *Pax7* promoter (Pax7-CreER^T2^). The model also contains a conditional oncogenic *Kras^G12D^* allele that is floxed, or preceded by a stop cassette flanked by loxp sites, at the endogenous *Kras* promoter (*LSL- Kras^G12D^*). Finally, the model has 2 copies of *Trp53* with floxed exons 2 through 10 (*P53^fl^*), and *Atrx* allele(s) with a floxed exon 18, which is an essential exon required for SWI/SNF protein function (*Atrx^fl^*). Because *Atrx* is X-linked, 2 *Atrx^fl^* alleles are required in female mice and 1 *Atrx^fl^* allele is required in male mice to generate tumors lacking *Atrx* expression. To initiate a sarcoma in the P7 KPA model (Figure 2A), we injected mice in the gastrocnemius muscle with 4-hydroxytamoxifen (4-OHT) to activate Cre recombinase in muscle satellite cells. Once activated, Cre drives excision of the stop cassette preceding oncogenic *Kras^G12D^* and leads to deletion of *Trp53* and *Atrx-*floxed alleles (Figure 2A). For all experiments, we used littermate control mice that retained at least 1 WT *Atrx* allele (P7 KP model, Figure 2A). Time-to-tumor detection following intramuscular 4-OHT injection was delayed in P7 KPA mice compared with P7 KP mice that retained *Atrx* expression. P7 KPA mice had a median of 55 days (range 27–77) compared with P7 KP mice, which had a median of 35 days (range 27–61) (Supplemental Figure 2A). The histologic appearance of P7 KPA sarcomas stained with H&E or myogenic markers was similar to P7 KP sarcomas, with most of the tumors mimicking human UPS (Figure 2B and Supplemental Figure 3), as previously described (23). A minority of the sarcomas in the mouse P7 KPA model were classified as rhabdomyosarcoma due to the presence of rhabdomyoblasts and myogenic markers (Figure 2C). There was a similar distribution of sarcoma subtypes identified within the P7 KP control cohort (Figure 2D). To assess *Atrx* recombination in the P7K KPA model, we generated cell lines from primary tumors. After culturing the cells in vitro for at least 8 passages to eliminate stromal cell contamination, we isolated genomic DNA and performed PCR genotyping for *Atrx*. As expected, the loxP-flanked exon 18 of *Atrx* was efficiently recombined in the P7 KPA model, which was confirmed by 2 genotyping assays (Figure 2E top and bottom).

### Atrx deletion increases sensitivity to DNA DSB-inducing therapies in vitro.

**Figure 3:**
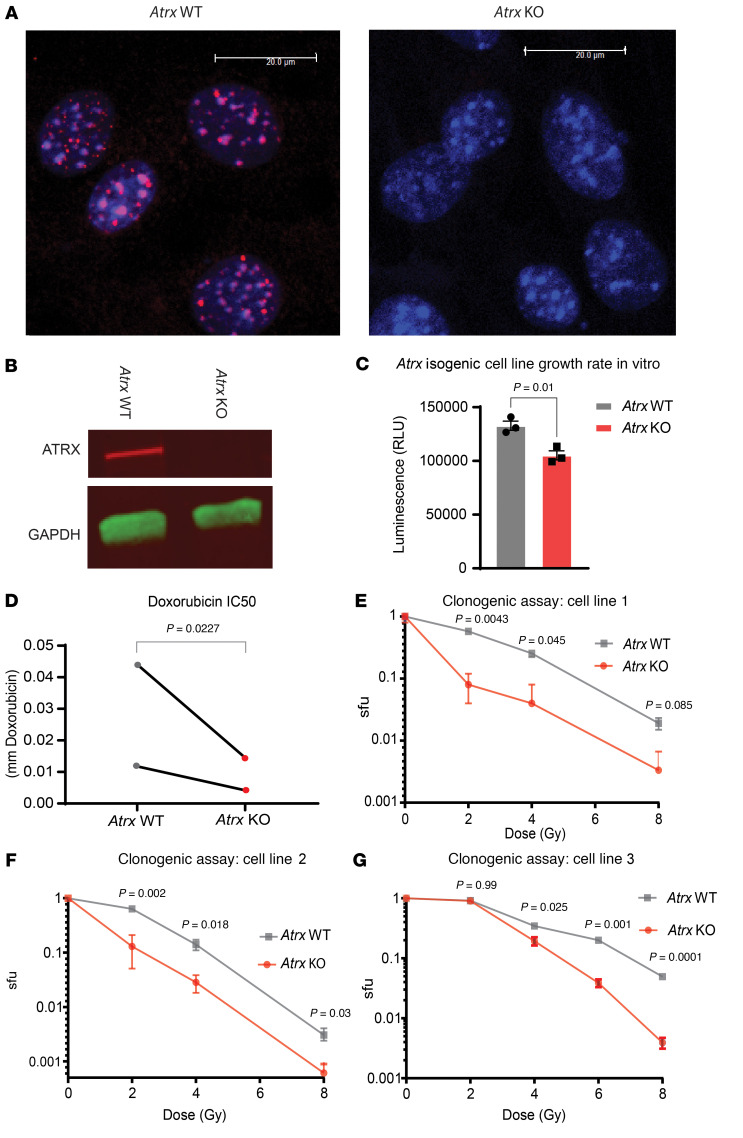
*Atrx* deletion increases sensitivity to DNA DSB-inducing therapeutics. (**A**) Immunofluorescence staining for ATRX (red) in isogenic cell lines derived from single cells after transfection with Cas9 and either a vector only control (left, “*Atrx* WT”), or a sgRNA to *Atrx* (right, “*Atrx* KO”). DAPI staining of nuclei in blue. Representative images, experimental validation of CRISPR mediated *Atrx* knockout repeated for each isogenic *Atrx* cell line pair used in this study. (**B**) Western blot of ATRX and GAPDH in *Atrx* WT and *Atrx* KO cell lines using LICOR Odyssey Imager with chemiluminescent secondary antibodies. Representative images, experimental validation of CRISPR mediated *Atrx* knockout repeated for each isogenic *Atrx* cell line pair used in this study. Colors are generated by fluorescent secondary antibodies, red bound to ATRX primary antibody, green bound to GAPDH primary antibody. (**C**) Growth assay for *Atrx* WT and *Atrx* KO cell lines, performed in triplicate and measured using Cell Titer Glo 2.0. Statistical analysis using unpaired 2-tailed *t* test**.** (**D**) IC_50_ assays in which *Atrx* isogenic cell line pairs (*n* = 2) were treated with the DNA DSB-inducing therapeutic doxorubicin. Statistical analysis was performed using a ratio paired *t* test. Each data point represents a biological replicate. (**E**–**G**) Clonogenic assay of isogenic *Atrx* deleted and intact cell line pairs after the indicated doses of ionizing radiation. Surviving fraction (sfu) is shown in log scale on the y-axis. Statistical analysis was performed using multiple Welch’s *t* tests corrected for multiple comparisons using the Holm-Šídák method. Each graph represents a separate biological replicate isogenic cell line pair.

To test how *Atrx* deletion impacted the chemotherapy response in vitro, we first generated murine UPS cell lines with activated oncogenic *Kras^G12D^* and *Trp53* deletion from KP mouse model sarcomas (24). We then used CRISPR-Cas9 gene editing to delete *Atrx* and used a vector-only control in this same cell line to generate an isogenic *Atrx* WT control. We confirmed successful KO of *Atrx* in the *Atrx* KO isogenic lines at the protein level (Figure 3, A and B). We also generated 2 human sarcoma cell lines from the 143B sarcoma parent line, 1 with CRISPR *ATRX* deletion and 1 without, using the identical procedure as above. Next, for our *Atrx* isogenic mouse sarcoma cell lines, we tested whether *Atrx* deletion affected cell line growth rate. Our results showed that *Atrx* deletion slowed cell line growth by a small, but statistically significant, amount (Figure 3C). We then tested whether *Atrx* deletion increased sensitivity to the DNA strand break inducing chemotherapy doxorubicin (Figure 3D). Since we observed an increased sensitivity to double strand break–inducing therapy, we reasoned that deletion of *Atrx* may also increase sensitivity to ionizing radiation in vitro. We performed clonogenic assays using single radiation doses (2 Gy to 8 Gy) and observed that *Atrx* deletion decreased sarcoma cell survival following irradiation in 3 different isogenic primary cell line pairs (Figure 3, E–G).

### Atrx deletion results in persistent DNA damage, telomere dysfunction–induced foci, and severe mitotic defects after irradiation.

**Figure 4:**
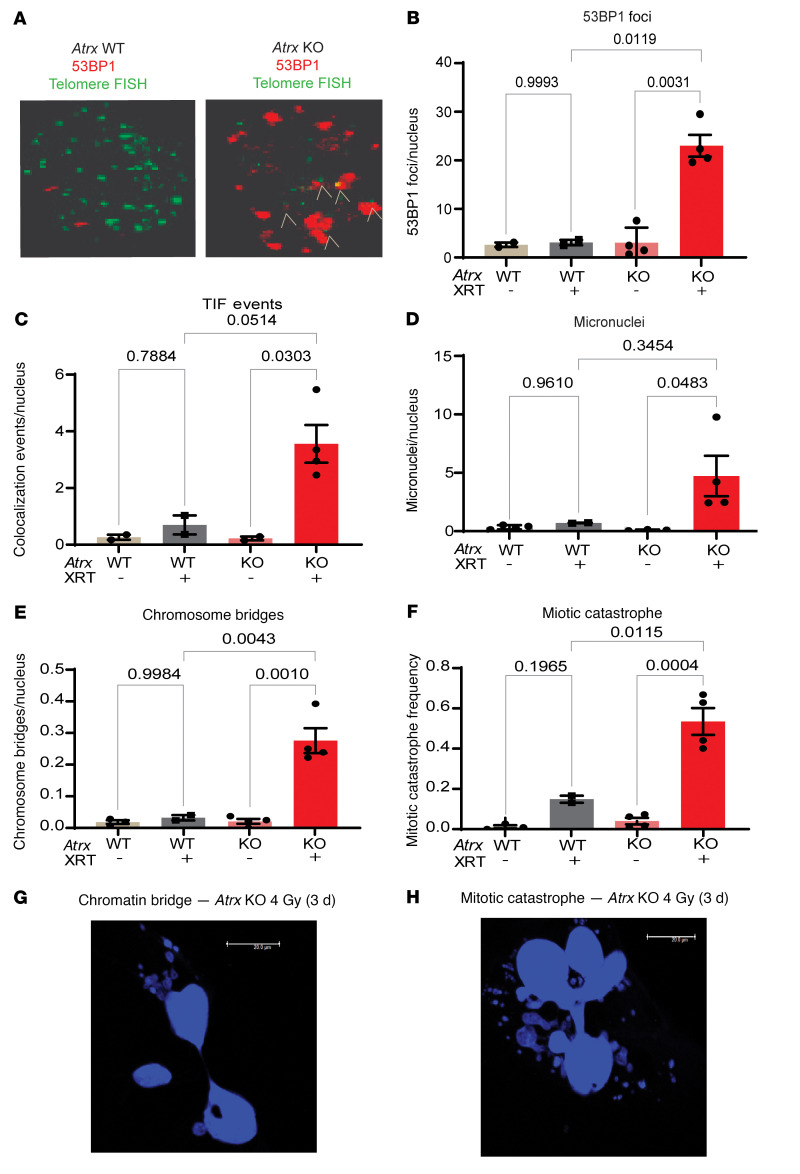
*Atrx* deletion leads to persistent DNA DSBs and telomere dysfunction after irradiation. (**A**) Representative images showing 53BP1 Foci (red) and telomere foci (green) in *Atrx* WT (left) and *Atrx* KO (right) cell lines 3 days after 4 Gy. White markers show colocalization of telomere FISH foci and 53BP1 foci, which are TIF. Representative images from experiments detailed in **C**. (**B**–**F**) Quantification of an *Atrx* isogenic cell line pair assayed 3 days after 4 Gy. Each dot represents an experimental repeat immunoFISH experiment of a single isogenic cell line pair with at least 7 images quantified for each experiment. Replicates are *Atrx* KO + IR (*n* = 4); *Atrx* KO untreated (*n* = 4); *Atrx* WT + IR (*n* = 2); and *Atrx* WT untreated (*n* = 3). Data are shown for 53BP1 foci (**B**), 53BP1 and telomere FISH colocalization marking TIFs (**C**), micronuclei (**D**), persistent chromosomal bridges between cells (**E**), and mitotic catastrophe (**F**). Statistical analysis was performed using a 2-way ANOVA with Tukey’s multiple comparisons test (**B**–**F**). (**G**) DAPI staining showing a chromatin bridge in an *Atrx* KO cell line treated with 4 Gy and assayed 3 days later. (**H**) DAPI staining showing a cell undergoing mitotic catastrophe in an *Atrx* KO cell line treated with 4 Gy and assayed 3 days later. Representative images from experiments detailed in **F**.

**Figure 5:**
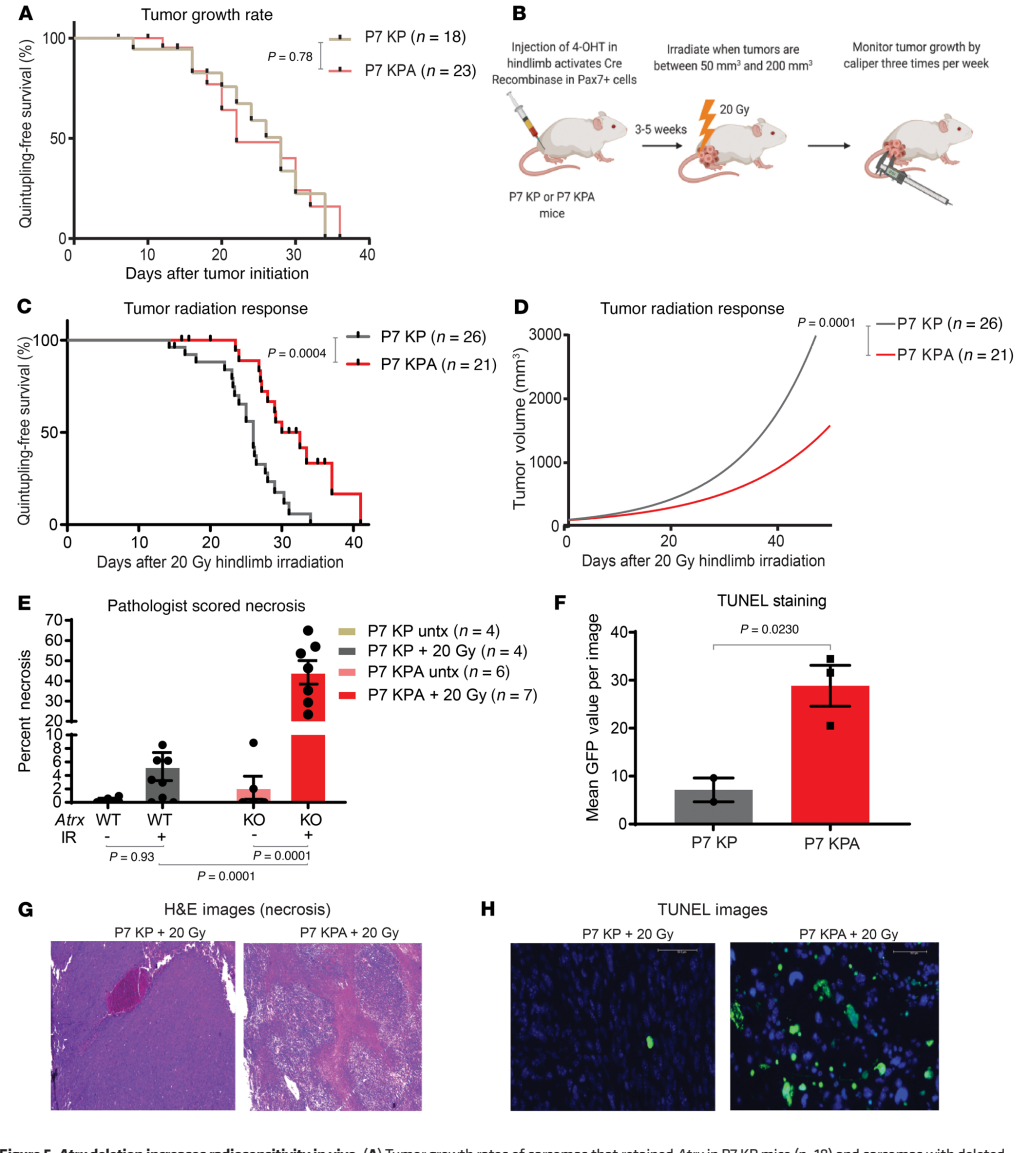
*Atrx* deletion increases radiosensitivity in vivo. (**A**) Tumor growth rates of sarcomas that retained *Atrx* in P7 KP mice (n=18) and sarcomas with deleted *Atrx* in P7 KPA mice (*n* = 23) as measured by time for tumor to quintuple in size relative to size at initial measurement. Comparison of survival curves was performed using log-rank (Mantel Cox) test. (**B**) Diagram summarizing experimental procedure for mouse sarcoma irradiation and monitoring. (**C**) Measurement of P7 KP (*n* = 26) and P7 KPA (*n* = 21) tumor growth rates after 20 Gy hindlimb irradiation, as measured by time for tumor to quintupling in size relative to size at treatment. Comparison of survival curves was performed using log-rank (Mantel Cox) test. (**D**) Nonlinear fit modeling of tumor growth curves for P7 KP (*n* = 26) and P7 KPA (*n* = 21) sarcomas after 20 Gy hindlimb irradiation. (**E**) Quantification of pathologist-scored percent area necrosis of samples that were either untreated (lighter colors) or treated with 20 Gy (darker colors) and harvested at 6 days. Each data point represents a biological replicate, and the number of samples for each arm is as annotated in figure legend, error bars showing SEM. Statistical analysis was performed using a 2-way ANOVA with Tukey’s multiple comparisons test. (**F**) Quantification of TUNEL positive fluorescence in frozen sections of P7 KP (*n* = 2) and P7 KPA (*n* = 3) tumor samples harvested 3 days after 20 Gy of hindlimb radiation. Each data point represents a biological replicate. Statistical comparison utilized an unpaired 2-tailed *t* test with Welch’s correction. (**G**) Representative H&E staining from sarcomas in P7 KP (left) and P7 KPA (right) mice harvested 6 days after 20 Gy. Images were scored for necrosis by a sarcoma pathologist. (**H**) Representative TUNEL staining from sarcomas from P7 KP (left) and P7 KPA (right) mice harvested 3 days after 20 Gy. GFP positive cells are TUNEL positive. Scale bar: 50 μm.

Next, we investigated the mechanism by which loss of *Atrx* increased radiosensitivity in our soft tissue sarcoma cell lines. Because of the DNA damage–associated signature we detected in *ATRX-*mutant human UPS (Supplemental Figure 1, B and C) and because of earlier reports describing a role for ATRX in DNA damage and telomere protection (21, 22), we hypothesized that deletion of *Atrx* would reduce DNA damage repair efficiency at telomeres, causing an increase in persistent DNA DSBs in these regions after irradiation. Persistent telomere breaks can lead to genomic instability, mitotic dysfunction, and cell death (25, 26). To test this hypothesis, we performed immunofluorescence staining for DNA DSB protein 53BP1 in conjunction with telomere fluorescence in situ hybridization (immunoFISH) in paired *Atrx* WT and *Atrx* KO isogenic cell lines 3 days after treatment with 4 Gy (Figure 4, A–F, and Supplemental Figure 4). This immunoFISH experiment demonstrated that *Atrx* deletion significantly increased 53BP1 foci 3 days after irradiation, suggesting a global increase in persistent DNA damage (Figure 4B). Additional analysis revealed that *Atrx* deletion increased the number of 53BP1 foci that colocalized with telomeres, also known as telomere dysfunction induced foci (TIF), suggesting that functional ATRX protects telomeres in sarcoma cells from radiation-induced DNA damage and TIFs (Figure 5, A and C).

**Figure 6:**
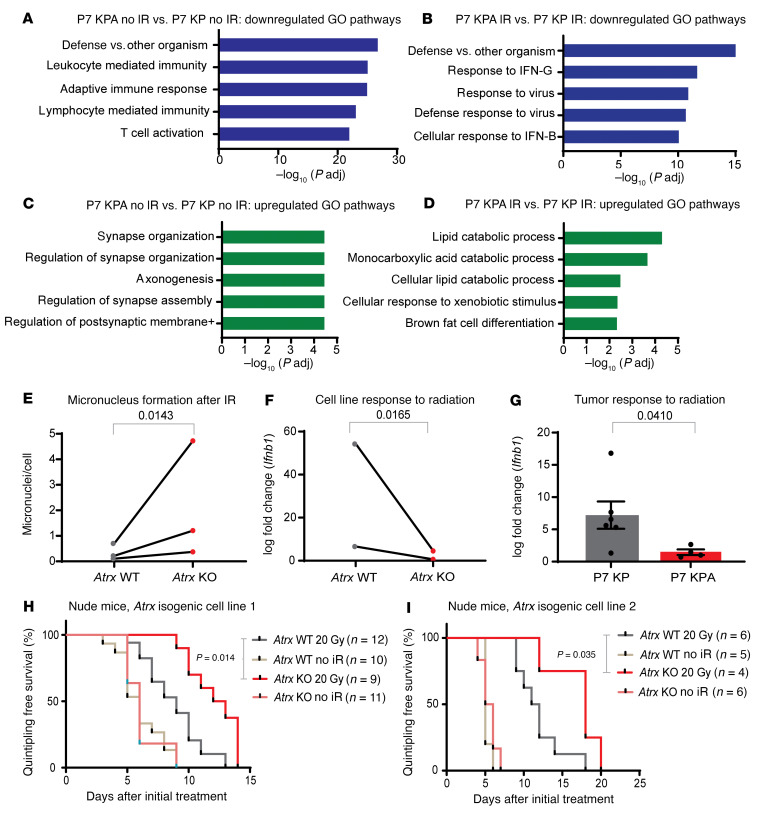
*Atrx* deletion radiosensitizes STS in a T cell–independent manner. (A)Top 5 downregulated pathways for RNA-Seq GO pathway enrichment analysis comparing unirradiated P7 KPA sarcomas (*n* = 4) and unirradiated P7 KP sarcomas (*n* = 4). (**B**) Top 5 downregulated pathways for RNA-Seq GO pathway enrichment analysis comparing P7 KPA sarcomas harvested 6 days after treatment with 20 Gy ionizing radiation (*n* = 4) and P7 KP sarcomas harvested 6 days after treatment with 20 Gy ionizing radiation (*n* = 8). (**C**) Top 5 upregulated pathways for RNA-Seq GO pathway enrichment analysis comparing unirradiated sarcomas in P7 KPA mice (*n* = 4) and unirradiated sarcomas in P7 KP mice (*n* = 4). (**D**) Top 5 upregulated pathways for RNA-Seq GO pathway enrichment analysis comparing sarcomas in P7 KPA mice harvested 6 days after treatment with 20 Gy ionizing radiation (IR) (*n* = 4) and sarcomas in P7 KP mice harvested 6 days after treatment with 20 Gy IR (*n* = 8). (**E**) Quantification of micronuclei for cell lines analyzed 3 days after treatment with 4 Gy. Each dot represents a biological replicate, and each biological replicate had at least 5 separate fields scored. For statistical analysis, a ratio paired 2-tailed *t* test was performed, pairing each *Atrx* KO cell line (*n* = 3) to its *Atrx* WT isogenic cell line (*n* = 3) counterpart. (**F**) RT-PCR quantification of log fold–change expression of *Ifnb1* of isogenic cell lines assayed 3 days after 4 Gy of ionizing radiation. For statistical analysis, a ratio paired *t* test was performed, pairing each *Atrx* KO cell line (*n* = 2) to its *Atrx* WT isogenic cell line (*n* = 2) counterpart. (**G**) RT-PCR quantification of log fold–change expression of *Ifnb1* of sarcomas from P7 KP (*n* = 6) and P7 KPA (*n* = 4) mice harvested 6 days after treatment with 20 Gy of ionizing radiation relative to its untreated control. Statistical comparison utilized an unpaired *t* test with Welch’s correction (**H** and **I**) A single cell line from 1 of 2 *Atrx* isogenic cell line pairs was implanted into the hindlimb muscle of nude T cell–deficient mice. Once tumors formed, mice were randomized to receive radiation therapy or no treatment. Number of tumors for each experimental arm are as shown in the figure legend. Survival curves were estimated for each group, considered separately, using the Kaplan-Meier method, and compared statistically using the log-rank (Mantel Cox) test.

As persistent DNA DSBs can lead to chromosome missegregation and mitotic error, we next evaluated whether irradiation of sarcoma cells with *Atrx* deletion caused increased mitotic dysfunction, including micronucleus formation, chromosome bridges, and mitotic catastrophe events. Quantification showed that the *Atrx* KO cell line had a significant increase in micronuclei after radiation, but we did not detect enhanced micronucleus formation in the *Atrx* WT cell line (Figure 4D). Irradiation of the *Atrx-*deleted sarcoma cell line also significantly increased chromosome bridge events and the number of cells undergoing mitotic catastrophe relative to its irradiated *Atrx* WT counterpart (Figure 4, E–H). Consistent with the enhanced mitotic dysfunction, immunofluorescence staining of the nuclear envelope protein Lamin B1 with DAPI counterstain showed that *Atrx* deletion increased micronuclei after treatment with ionizing radiation across multiple isogenic cell line pairs (Figure 6E and Supplemental Figure 4, D and E).

### Atrx deletion radiosensitizes and increases cell death in sarcomas in autochthonous primary mouse models.

Next, we set out to evaluate whether *Atrx* deletion increased tumor response to radiation therapy in the primary P7 KP mouse model. Loss of *Atrx* had no discernable effect on sarcoma growth rates in the absence of treatment, but following a single dose of 20 Gy focal radiation therapy, tumors in P7 KPA mice demonstrated a significant growth delay relative to tumors in the P7 KP mice (Figure 5, A–D, and Supplemental Figure 8, B and C). In a separate cohort of these mice, we harvested sarcomas in P7 KPA and P7 KP mice 6 days after a single fraction of 20 Gy focal irradiation. Examination of H&E stained sections from these tumors revealed that *Atrx* deletion resulted in a significant increase in tumor necrosis 6 days after irradiation (Figure 5, E and G). In addition, deletion of *Atrx* in sarcomas harvested 3 days after 20 Gy focal irradiation led to a significant increase in cell death detected through TUNEL staining (Figure 5, F and H). P7 KPA tumors also had a significantly lower fraction of Ki67 positive cells by IHC 6 days after radiation therapy (Supplemental Figure 4, A and B). To evaluate whether this phenotype was limited to the *Kras^G12D^* expressing P7 KP model, we then repeated this experiment in a second primary mouse model of soft tissue sarcoma. The P7 P + MCA model does not utilize a genetically engineered conditional oncogenic *Kras^G12D^* allele, but instead is initiated by intramuscular injection of 4-OHT in *Pax7-CreER^T2^*; *Trp53^fl/fl^* mice to delete *Trp53*, followed by injection of 3-methylcholanthrene (3-MCA), a potent carcinogen that drives base substitutions at the site of injection (Supplemental Figure 5A) (27). Similar to the P7 KPA model, *Atrx* deletion in the P7 P + MCA model significantly delayed tumor growth after radiation but this growth reduction was not observed in unirradiated cohorts (Supplemental Figure 4C and Supplemental Figure 8, E–G). Therefore, deletion of *Atrx* caused radiosensitivity in 2 independent primary mouse models of soft tissue sarcoma.

### ATRX-deleted sarcomas have reduced adaptive and innate immune signaling after ionizing radiation.

We then evaluated whether the observed increase in radiosensitivity in vivo after *Atrx* deletion was mediated by the adaptive immune system. To test this, we first performed RNA-Seq of cohorts of P7 KPA and P7 KP sarcomas that were not irradiated. Both gene ontology (GO) (Figure 6A) and KEGG pathway enrichment analysis (Supplemental Figure 6A) of these samples revealed a marked downregulation of adaptive immune signaling in the P7 KPA cohort compared with the P7 KP control. Upregulated pathways in this pathway analysis of unirradiated tumors had a weaker association and were primarily associated with neuronal organization (Figure 6C). We then performed RNA-Seq of P7 KPA and P7 KP tumors harvested 6 days after 20 Gy of radiation therapy. GO pathway enrichment analysis of these tumors also demonstrated a marked downregulation of adaptive immune signaling (Figure 6B). Both cellular response to interferon-β and defense response to virus were among the most highly enriched pathways in our analysis of downregulated gene sets in irradiated P7 KPA tumors compared with the irradiated P7 KP controls. Interestingly, upregulated pathways after irradiation for P7 KPA tumors relative to P7 KP control were related to lipid catabolism and brown fat cell differentiation (Figure 6D). Tying together the upregulated and downregulated pathways enriched in ATRX-deleted tumors after ionizing radiation, the CGAS/STING pathway is an innate immune pathway that detects dsDNA in the cytoplasm and activates type-I interferon signaling (28). Recent research has also demonstrated that CGAS/STING signaling can negatively regulate thermogenesis and lipid catabolism (29–31).

### Atrx deletion impairs type-I IFN response after radiation.

When comparing irradiated P7 KPA and P7 KP tumors, we noted that, in addition to downregulation of adaptive immune signaling, there was a significant downregulation of antiviral defense and type-I IFN signaling–related pathways (Figure 6B). In the context of radiation therapy, one prominent mechanism for activation of type-I IFN signaling is the CGAS/STING pathway.

Micronuclei can rupture, releasing DS DNA into the cytoplasm that activates the cytoplasmic DS DNA sensing system, CGAS/STING, to drive expression of type-I IFN (18). Because *Atrx*-deleted sarcoma cells had increased micronuclei after irradiation (Figure 6E), we next tested whether *Atrx* deletion increased IFN-β (*Ifnb1*) expression after ionizing radiation in vitro. Interestingly, we found that *Atrx* deletion reduced *Ifnb1* expression following 4 Gy relative to the matched isogenic *Atrx* WT control (Figure 6F). Because this reduction in Ifnb1 expression in ATRX-deleted cell lines could, in part, be explained by the 20% reduction in growth rate in vitro after ATRX deletion (Figure 3C), we next tested whether *Ifnb1* expression would also be reduced in ATRX-deleted sarcomas in vivo, where no significant difference in growth rates was observed. To this end we collected P7 KPA or P7 KPA tumors 6 days after treatment with 20 Gy and analyzed their *Ifnb1* expression. We found that, in the setting of in vivo irradiation, *Atrx* deletion also impaired type-I IFN signaling (Figure 6G). Because *Atrx* deletion radiosensitized cell lines in vitro and reduced type-I IFN signaling in vitro, we next questioned whether *Atrx* deletion would sensitize in vivo sarcomas in a T cell–independent manner. To study this question, we transplanted isogenic KP sarcoma cell lines with and without *Atrx* deletion into the hindlimb muscles of athymic nude mice and measured the rate of tumor growth (Supplemental Figure 5B). We found that *Atrx* deletion radiosensitized the transplanted tumors even in the absence of an intact immune system (Figure 6, H and I, and Supplemental Figure 8, H–J). Together, these results showed that *Atrx* deletion increased radiosensitivity in both T cell–deficient and immunocompetent models in vivo.

### Atrx deletion impairs CGAS/STING signaling in soft tissue sarcomas.

**Figure 7:**
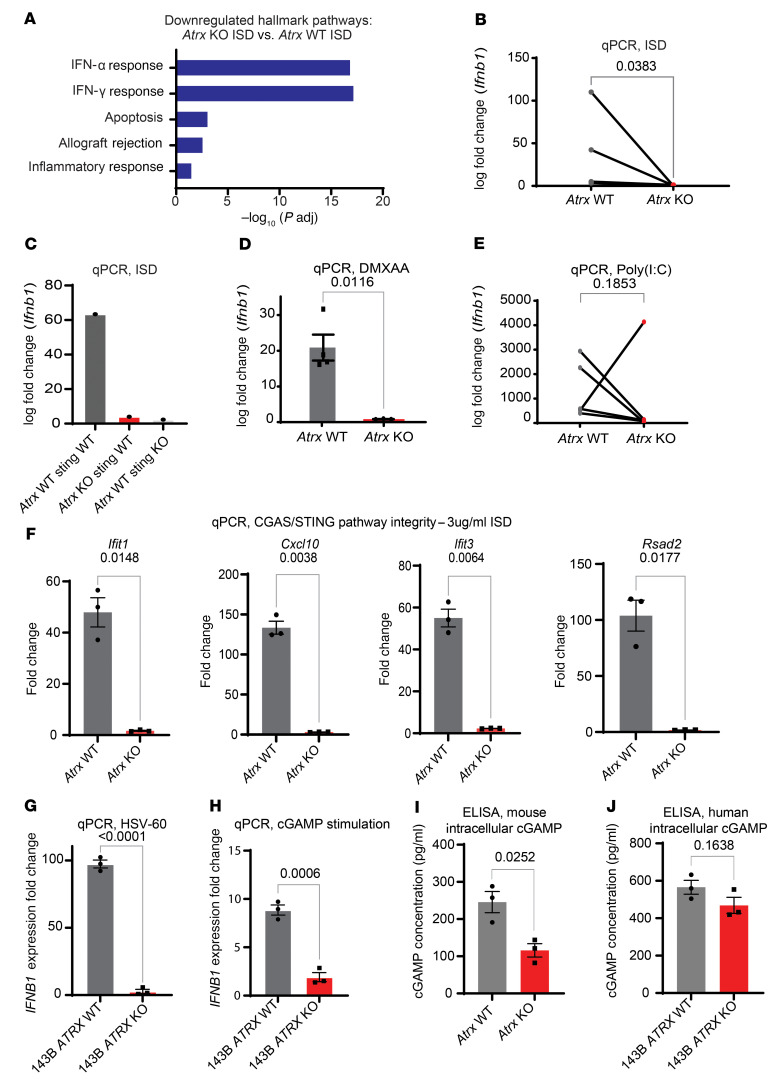
Atrx deletion suppresses type I IFN signaling in a CGAS/STING-dependent manner. (**A**) Hallmark pathway gene set enrichment analysis of RNA-Seq data comparing 3 *Atrx* KO isogenic cell lines treated with ISD to 3 *Atrx* WT isogenic cell lines treated with ISD. (**B**) RT-PCR of *Ifnb1* for *Atrx* WT (*n* = 3) and *Atrx* KO (*n* = 3) isogenic cell line pairs transfected with ISD at a concentration of 3 μg/mL and harvested 24 hours after treatment. Statistical analysis was performed using a ratio paired *t* test. All data points represent biological replicates. (**C**) RT-PCR quantification of *Ifnb1* expression after ISD stimulation for an *Atrx* WT and *Sting* WT sarcoma cell line without or with CRISPR-mediated knockout of *Atrx* alone or *Sting* alone. These experiments were performed using 3 technical replicates per experimental condition and the resulting log fold–change value is shown. (**D**) RT-PCR of *Ifnb1* for *Atrx* isogenic cell line pair 1 treated for 24 hours with 100 μg/mL DMXAA, a potent STING agonist and cGAMP analogue. Each dot represents a separate experimental repeat of the RT-PCR assay (*n* = 4 per arm). Statistical analysis was performed using an unpaired *t* test with Welch’s correction. (**E**) RT-PCR of *Ifnb1* for *Atrx* isogenic cell line pairs (*n* = 4) treated with Poly(I:C) at a concentration of 1 μg/mL and harvested at 24 hours. Statistical analysis was performed using a ratio paired *t* test. All data points represent biological replicates. (**F**) RT-PCR quantification of 4 other ISGs downstream of the CGAS pathway for *Atrx* isogenic cell line pairs (*n* = 3) harvested 24 hours after transfection with ISD. Statistical analysis was performed using an unpaired *t* test with Welch’s correction. (**G**) RT-PCR quantification of *IFNB1* for the 143B human sarcoma cell line with or without *ATRX* KO harvested 24 hours after treatment with oncolytic herpesvirus HSV-60 DNA. Each dot represents a separate experimental repeat of the RT-PCR assay (*n* = 3 per arm). Statistical analysis was performed using an unpaired *t* test with Welch’s correction. (**H**) RT-PCR of *IFNB1* for 143B human sarcoma cell line with or without *ATRX* KO treated for 24 hours with 100 μg/mL cGAMP. Each dot represents a separate experimental repeat of the RT-PCR assay (*n* = 3 per arm). Statistical analysis was performed using an unpaired *t* test with Welch’s correction. (**I** and **J**) ELISA for cGAMP was performed using either mouse (**I**) or human (**J**) ATRX isogenic cell lines in triplicate. Raw values were fit to a standard curve with the use of the ELISATriple Plate software from Cayman. Each dot represents a technical replicate of the experiment, error bars showing SEM. Statistical analysis was performed using an unpaired *t* test with Welch’s correction for both **I** and **J**.

To determine whether the observed reduction in *Ifnb1* expression after radiation in our ATRX-deleted sarcomas was the result of an impairment in the CGAS/STING pathway, we first assessed the proficiency of the pathway in mouse sarcoma cell lines via CGAS/STING induction using interferon stimulatory DNA (ISD). ISD is 45-bp non-CpG oligomer derived from the *Listeria monocytogenes* genome that is known to induce CGAS/STING activation (32). First, we performed RNA-Seq on 3 *Atrx* intact and deficient isogenic sarcoma cell line pairs after treatment with either ISD or untreated controls. Pathway analysis revealed a significant downregulation of type-I IFN signaling in *Atrx-*deficient cell lines relative to their *Atrx*-intact controls after treatment with ISD (Figure 7A). We then performed a series of qPCR experiments to confirm downregulation of the CGAS/STING pathway in *Atrx*-deleted sarcomas. In 4 different P7 KP tumor-derived isogenic cell lines with or without *Atrx* deletion, we found that after transfections with ISD, cells with *Atrx* deletion had markedly decreased CGAS/STING–mediated *Ifnb1* expression (Figure 7B). We then performed a similar ISD stimulation experiment with P7 KP–derived isogenic cell lines with CRISPR-mediated genetic knockout of *Atrx* or *Atrx* and *Sting* combined. Our results in this experiment showed that *Atrx* deletion alone reduced CGAS/STING–mediated *IfnB1* expression to a level similar to that seen with a combined *Sting*-and-*Atrx* KO cell line (Figure 7C).

In order to ascertain what part of the CGAS/STING signaling pathway was impaired following deletion of *Atrx*, we next treated the isogenic cell lines with DMXAA, a cGAMP analogue and STING agonist. DMXAA treatment induced significantly less *Ifnb1* expression in *Atrx-*deleted cell lines (Figure 7D), suggesting that part of the observed impairment in CGAS/STING signaling was due to regulation of pathway components downstream of CGAS or cGAMP. An alternative explanation for these results is that loss of ATRX function disrupts *Ifnb1* gene transcription independent of CGAS/STING signaling. Therefore, we treated cell lines with and without *Atrx* deletion with Poly(I:C), an RNA-based compound known to stimulate *Ifnb1* via a CGAS/STING–independent pathway. Stimulation with Poly(I:C) induced greater than 25-fold increase in *Infb1* expression relative to untreated controls in every *Atrx* intact and deleted cell line tested (Figure 7E). To validate our RNA-Seq findings that *Atrx* deletion downregulated type I–IFN response and to ensure that the effect was not specific to *Ifnb1* alone, we performed qPCR to asses the levels of 4 IFN-stimulatory genes (*Ifit1, Cxcl10, Ifit3,* and *Rsad2*) after stimulation with ISD (Figure 7F). Our results showed that after ISD treatment these 4 genes were significantly reduced in *Atrx*-deleted cell lines. To test whether these findings also held true in human sarcomas, we used our isogenic 143B human sarcoma cell line with or without CRISPR *ATRX* knockout and assessed *IFNB1* expression levels after treatment with oHSV-60, a 60-bp DNA sequence known to potently stimulate the CGAS/STING pathway. Our results from this experiment showed that 143B *ATRX* KO cell lines had significantly less *IFNB1* expression by qPCR than their 143B *ATRX* WT counterparts (Figure 7G). We also stimulated the 143B isogenic cell lines with cGAMP and found that the 143B *ATRX* KO cell line had significantly less expression of *IFNB1*, suggesting that at least part of the CGAS/STING pathway impairment was occurring downstream of CGAS in our human sarcoma cell lines (Figure 7H). Finally, we decided to test whether *ATRX* KO resulted in reduction in cGAMP in our mouse and human (Figure 7J) cell lines. Our findings demonstrated that *ATRX* KO in our mouse sarcoma cell lines resulted in a significant reduction in intracellular cGAMP (Figure 7I). The reduction in human 143B *ATRX* KO cell lines, however, did not reach statistical significance (Figure 7J).

Next, because ATRX is an epigenetic regulator known to affect transcriptional levels of multiple genes (33, 34), we questioned whether deletion of *Atrx* may be decreasing expression of key CGAS/STING pathway components. Analysis of our RNA-Seq data revealed that no major pathway components, including *Cgas*, *Sting*, *Irf3,* or *Tbk1*, were downregulated in *Atrx*-deleted mouse sarcomas relative to their *Atrx* WT control either in vitro or in vivo (Supplemental Figure 6B). We then hypothesized that *Atrx* deletion and the associated increase in micronuclei and persistent DNA damage may create a chronic inflammatory state that constitutes a selective pressure leading to acquired mutations in the CGAS/STING pathway. To test this, we performed tumor-normal paired whole exome sequencing on both P7 KPA tumor and P7 KP tumors. Our results showed no recurrent mutations that are, to the best of our knowledge, associated with the CGAS/STING pathway (Supplemental Figures 9 and 10). These whole exome sequencing results suggested that the downregulation of CGAS/STING signaling we observed was not mediated by mutations in the CGAS/STING signaling pathway. Together these data demonstrated that *Atrx* deletion impaired response to the CGAS/STING pathway in soft tissue sarcoma. Our studies further show that this *Atrx*-mediated downregulation can exist both at the level of cGAMP and also separately, at or downstream of STING. Finally, we demonstrated that this downregulation of CGAS/STING signaling did not occur via selective pressure for mutation of CGAS/STING pathway genes and was not driven by transcriptional downregulation of CGAS/STING pathway components.

### Deletion of Atrx increases sensitivity to oncolytic herpesvirus therapy.

**Figure 8:**
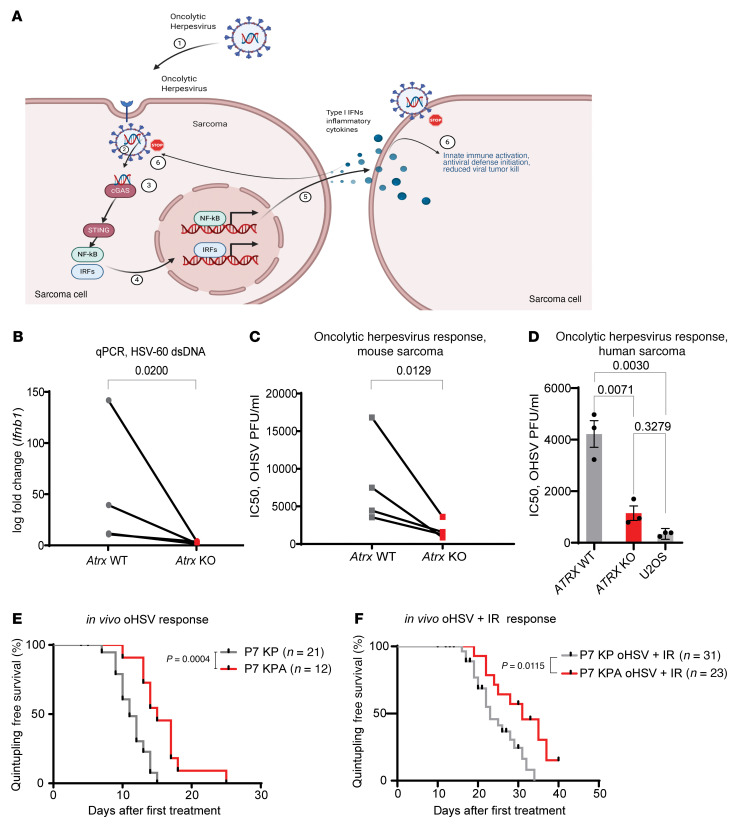
Atrx deletion increases sarcoma sensitivity to oncolytic herpesvirus therapy. (**A**) Diagram showing the interaction between oncolytic herpesvirus and CGAS/STING pathway in *Atrx* WT sarcoma cells. Oncolytic herpes virus dsDNA is detected and CGAS/STING signaling activates the innate immune response, which inhibits viral spread and oncolytic herpesvirus efficacy. (**B**) RT-PCR of *Ifnb1* for *Atrx* WT and *Atrx* KO isogenic sarcoma cell lines (*n* = 3) assayed 24 hours after treatment with HSV-60, a 60-bp dsDNA oligonucleotide derived from HSV-1 that is a known inducer of CGAS/STING signaling. For statistical analysis, a ratio paired *t* test was performed, pairing each *Atrx* KO cell line to its *Atrx* WT isogenic cell line counterpart. (**C**) IC_50_ assays for isogenic mouse sarcoma cell lines (*n* = 3) treated with oncolytic herpesvirus (oHSV) ordered from Imanis Life Sciences. Differences between all paired cell lines are statistically significant (*P* <.05) as analyzed by a ratio paired *t* test. Each data point represents a biological replicate. (**D**) IC_50_ assays for the 143B human Sarcoma cell line with or without *ATRX* deletion, as well as the U2OS human sarcoma cell line, which is lacks protein expression of both ATRX and STING. Each dot represents a separate experimental replicate of the IC_50_ assay (*n* = 3 per cell line). Statistical analysis was performed using multiple Welch’s *t* tests corrected for multiple comparisons using the Holm-Šídák method. (**E**) Measurement of P7 KP (*n* = 21) and P7KPA (*n* = 12) tumor growth after treatment with a mouse optimized version of TVEC oncolytic herpesvirus, as measured by time for tumor to quintuple in size relative to size at treatment. Comparison of survival curves was performed using log-rank (Mantel Cox) test. (**F**) Measurement of P7 KP (*n* = 31) and P7 KPA (*n* = 23) tumor growth rates after 20 Gy hindlimb irradiation followed by treatment with a mouse optimized TVEC oncolytic herpesvirus, as measured by time for tumor to quintuple in size relative to size at treatment. Comparison of survival curves was performed using log-rank (Mantel Cox) test.

Activation of CGAS/STING in response to viral dsDNA led to expression of type I–IFN inflammatory cytokines. This induction of type I–IFN signaling, in turn, limits viral replication and spread of herpes simplex virus type 1 (HSV-1) and herpesvirus based oncolytic viral cancer therapy (Figure 8A) (32). KEGG pathway enrichment analysis of RNA-Seq data revealed a marked downregulation of the HSV-1 response pathway in P7 KPA tumors relative to P7 KP controls (Supplemental Figure 6, A and C). Based on this downregulation and the finding that *Atrx* deletion reduced activation of the CGAS/STING response to microbial-derived dsDNA, we investigated whether *Atrx* deletion increased sensitivity to an oncolytic HSV-1 designed to specifically target and kill tumor cells. We first tested the effect of transfection of dsDNA from HSV type I (HSV-60), a well described stimulant of CGAS/STING signaling, in 3 isogenic mouse sarcoma cell line pairs. There was significantly less induction of type I–IFN expression in the *Atrx* KO cell lines after HSV-60 stimulation when compared with the *Atrx* WT cell lines (Figure 8B). We therefore tested the impact of *Atrx* deletion on sarcoma cell infection with an oncolytic herpesvirus variant of HSV-1 ordered from Imanis Life Sciences (Imanis oHSV) that has been modified to specifically target and destroy tumor cells (35). The Imanis oHSV used is identical to the variant described above (35), with the exception that it contains no transgenic insertion between the GFP and CMV sites. Other variants, including the G207 oHSV which is closely related to the virus used for this study, are currently in phase II clinical trials for the treatment of recurrent supratentorial brain tumors (NCT03911388, NCT02457845) (36). To assess whether deletion of *Atrx* sensitized sarcoma cells to oHSV, we performed an IC_50_ assay using 4 different isogenic cell line pairs. We found that *Atrx* deletion significantly increased sensitivity to Imanis oHSV in *Atrx-*deficient sarcoma cell lines for every isogenic pair tested (Figure 8C). To extend these data, we performed clonogenic assays in which we treated both the *Atrx* WT and *Atrx* KO cell lines with an identical concentration of Imanis oHSV. We observed a significant reduction in the number of colonies formed in the *Atrx* KO cell lines (Supplemental Figure 7, A and B).

Based on these results, we obtained from Amgen a variant of talimogene laherparepvec (TVEC), an oncolytic herpesvirus variant with significant translational potential. In 2015, the FDA approved TVEC for the treatment of advanced melanoma (37). This variant of TVEC is identical to that used in clinical practice except that it expresses mouse granulocyte macrophage colony-stimulating factor (GM-CSF) rather than human GM-CSF. All oncolytic herpesvirus experiments performed from this point on were completed using TVEC. First, we tested whether *ATRX* deletion also increased sensitivity in vitro to TVEC in the human 143B sarcoma cell line. Consistent with our findings in mouse sarcoma cells, the 143B *ATRX* KO cells were significantly more sensitive to oncolytic herpesvirus than the *ATRX* WT isogenic counterpart (Figure 8D, left and middle bars). Interestingly, we also observed that, after TVEC treatment, there was no significant difference in IC_50_ values between the 143B *ATRX* KO cell lines and the control U2OS human sarcoma cell line known to lack STING protein expression (Figure 8D, middle and right bars). Next, we tested the effect of *Atrx* deletion on TVEC sensitivity in vivo using the autochthonous P7 KPA and P7 KP models. In the *Atrx*-deleted P7 KPA model, sarcomas had significantly slower growth after treatment with TVEC compared with the P7 KP (*Atrx* intact) control (Figure 8E). Moreover, primary sarcomas in the P7 KPA model treated with radiotherapy followed by TVEC also showed a significant growth delay relative to controls in the P7 KP mice, though this effect was additive rather than synergistic (Figure 8F). Together, these findings demonstrate that *Atrx* deletion impaired CGAS/STING response to HSV-1 dsDNA and increased susceptibility to oncolytic herpesvirus therapy in multiple human and mouse models of soft tissue sarcoma.

## Discussion

The contribution of *ATRX* loss to tumor development and treatment response is not well defined, despite mutations in *ATRX* occurring in approximately 6% of all human cancers (4). Using a mouse model of soft tissue sarcoma, we identified 2 categories of therapeutic vulnerability in tumors lacking *Atrx* function. Specifically, we found that *Atrx* loss-of-function mutations in soft tissue sarcoma confer sensitivity to established therapies, such as radiation therapy, as well as emerging therapies. We further showed that, in both human and mouse sarcoma models, *Atrx* loss-of-function mutation led to a deficiency in CGAS/STING signaling that could be therapeutically targeted by a mouse-specific variant of the FDA-approved oncolytic herpesvirus TVEC.

Here, we developed a primary mouse model that recapitulates some of the most frequently occurring hallmarks of human undifferentiated pleomorphic soft tissue sarcoma: loss of TP53 function, upregulated RAS pathway signaling, and loss of *ATRX* (4, 38, 39). A key strength of this sarcoma model is that tumors arise in a spatially and temporally restricted manner and in a native microenvironment with an intact immune system. Our genetic experiments demonstrated that loss of *Atrx* increased radiosensitivity in 4 model systems: sarcoma cells in vitro, sarcoma cells transplanted into immunodeficient mice in vivo, primary *Kras^G12D^; Trp53^–/–^* sarcomas in P7 KP mice, and primary *Trp53^–/–^* + MCA sarcomas in P7 P mice. Therefore, our findings suggest that *ATRX* mutations may be used to identify a subset of soft tissue sarcomas and potentially other tumors that are more sensitive to radiation therapy in the clinical setting.

Mechanistically, our work indicates that loss-of-function mutations in *Atrx* drive an increase in persistent DNA damage at telomeres after ionizing radiation therapy. This finding is consistent with previous reports suggesting that ATRX impairs DNA damage repair and increases telomere sister chromatid cohesion defects after induction of telomere DSBs (10, 11, 14, 21, 40). While this defect was suggested by others to recapitulate key hallmarks of ALT-positive cancers, our study also links the increased propensity for TIFs in *Atrx-*deleted sarcomas to increased mitotic catastrophe and necrosis after treatment with radiation therapy. Together, these findings define a mechanism for how *Atrx* deletion increases radiosensitivity. We further extended these findings by demonstrating that *Atrx* deletion decreased adaptive immune signaling and interferon signaling after ionizing radiation in vivo. We then showed that loss of *Atrx* can radiosensitize soft tissue sarcomas in a T cell independent manner in vivo, as demonstrated by transplant experiments in immunodeficient nude mice.

DNA damage–induced mitotic dysfunction led to increased micronuclei that frequently ruptured, releasing dsDNA into the cytoplasm. This dsDNA can activate CGAS/STING signaling, which thereby increased innate and adaptive immune signaling in tumors (41). The CGAS/STING pathway is emerging as a key regulator of cancer development and therapeutic response with roles in tumor development, metastasis, immunomodulation, senescence, viral defense, and autophagy (42–48). Previous work demonstrated that knockdown of Atrx or its partners Daxx and H3.3 inhibited CGAS/STING signaling in response to high levels of telomeric dsDNA in the cytoplasm of ALT-positive cell lines (15). It was unclear, however, whether impaired CGAS/STING signaling would be present in *Atrx*-deleted sarcomas and how radiation would alter this pathway in vitro and in vivo.

Our findings demonstrate that *Atrx* deletion in both human and mouse sarcomas impaired CGAS/STING signaling response to dsDNA transfection in vitro and to radiation in vitro and in vivo. We observed that *Atrx* deletion resulted in downregulation of CGAS/STING signaling and the CGAS/STING pathway. This signaling deficit occurred at multiple independent points within the CGAS/STING pathway. First, in mouse sarcoma cell lines lacking *Atrx,* we observed a decrease in intracellular cGAMP, a key messenger that activates STING signaling. Additionally, after transfection of human and mouse sarcoma cell lines with artificially produced cGAMP or cGAMP analogues like DMXAA, in cells lacking *Atrx,* we identified a second deficiency in the CGAS/STING pathway that can be localized to a pathway component between STING and IFNB1 in the signaling cascade.

Downregulation of cGAMP production in *Atrx*-deleted sarcomas could potentially be a result of decreased expression of genes in the STING signaling pathway. As a known epigenetic regulator, we therefore hypothesized that ATRX deletion may alter CGAS/STING signaling via reduction of expression of key CGAS/STING pathway genes. Our RNA-Seq results, however, showed that ATRX deletion did not result in a decrease in the expression of CGAS/STING pathway genes, indicating that the downregulation of cGAMP production is not mediated through direct transcriptional regulation. We then tested whether *Atrx* deletion created a cellular environment or selective pressure in sarcomas favoring the accumulation of acquired mutations in the CGAS/STING pathway. To investigate this possibility, we performed whole exome sequencing of sarcoma and normal matched samples in P7 KPA and P7 KP mice. However, our genomic sequencing analysis failed to identify any mutations in either CGAS/STING signaling genes or in other genes known to regulate this pathway. Together, these results indicate that the effect of *Atrx* deletion on CGAS/STING signaling was not mediated via transcriptional regulation or via selective pressures leading to CGAS/STING pathway mutations.

One alternative explanation for the downregulation of cGAMP production is that *Atrx* deletion could regulate CGAS/STING signaling through a posttranslational mechanism. While beyond the scope of the current study, future studies can investigate this possibility. Specifically, a key area for future investigation will be regulation of STING trafficking, as STING translocation from the ER to the Golgi is required for downstream signaling. Interestingly, one study has identified MRE11 as playing a key role in the process of STING translocation (49). A second possible explanation could be related to STING’s emerging role in the inhibition of fat thermogenesis and decreased production of polyunsaturated fatty acids. Recently published work demonstrated that STING ablation inhibits FADS2, the rate-limiting enzyme in polyunsaturated fatty acid desaturation, while polyunsaturated fatty acids, in turn, inhibited STING signaling (29). In our study, GO pathway enrichment analysis of RNA-Seq did show an upregulation of pathways related to brown fat cell differentiation and lipid catabolism in ATRX-deleted sarcomas. Taken together, these results provide a foundation for future studies to investigate the relationship between ATRX, lipid catabolism, and STING inhibition.

Importantly, a key function of the CGAS/STING pathway in normal cells is innate immune defense against dsDNA viruses. It has been previously demonstrated that deletion or impairment of the CGAS/STING pathway can increase susceptibility to oncolytic herpesvirus in vitro and in vivo in both normal cells and cancer (28, 48, 50–53). Our work using both mouse and human sarcoma models extends these findings by showing that *Atrx* deletion impairs the CGAS/STING pathway and increases sarcoma susceptibility to oncolytic herpesvirus in vitro and in vivo. ATRX may also contribute to dsDNA antiviral immunity via alterative mechanisms such as maintenance of viral heterochromatin (54). Future studies will be needed to determine whether ATRX-mediated effects on viral heterochromatin also contribute to the sensitivity to oncolytic herpesvirus observed in *Atrx*-deleted sarcoma cells. Regardless of which additional mechanisms are involved, our finding that *Atrx* deletion increased soft tissue sarcoma susceptibility to dsDNA oncolytic herpesvirus in vitro suggests that patients with sarcomas and perhaps other tumors with *ATRX* loss-of-function mutations may benefit from oncolytic virus (OV) therapy. OV therapy is an emerging cancer therapy, utilized in more than 90 recent clinical trials (55). Most of the trials underway are utilizing either oncolytic adenovirus, herpesvirus, polio virus, or vaccinia virus (37, 56, 57). In fact, talimogene laherparepvec (T-VEC), an oncolytic herpesvirus similar to the oncolytic herpesvirus used in this study, was the first OV therapy approved by the FDA to treat unresectable metastatic melanoma (57, 58). Because deletion of *Atrx* sensitizes sarcoma cells to oncolytic herpes virus and radiation therapy, tumors with loss-of-function mutations in *ATRX* may be particularly sensitive to the combination of oncolytic herpesvirus with radiation therapy. In addition, a recently completed phase II clinical trial evaluating a PD-1 immune checkpoint inhibitor and T-VEC oncolytic herpesvirus combination therapy in advanced sarcoma showed a promising overall objective response rate of 35% (59). We believe that our work lays the foundation for future studies examining the effect of ATRX loss on the response of soft tissue sarcomas to therapeutic combinations of PD-1 inhibitors, oHSV, and radiation therapy.

Collectively, these results show that loss of ATRX function impairs the CGAS/STING signaling pathway and promotes the response to radiation therapy and oncolytic virus therapy in soft tissue sarcoma. These findings suggest that, for these cancer therapies, *ATRX* mutation status in sarcomas and perhaps other cancers may be a biomarker for treatment response.

## Methods

### Experimental models and subject details

#### Mouse strains.

Mouse strains used in the primary mouse model were on a mixed 129S_4_/SvJae and C57/Bl6 background. *LSL-Kras^G12D^* mice were provided by Tyler Jacks (Massachusetts Institute of Technology, Cambridge, Massachusetts, USA) (60). *Trp53^Fl^* mice were provided by Anton Berns (Netherlands Cancer Institute, Amsterdam, Netherlands) (61). *Atrx^Fl^* mice were provided by Richard Gibbons (University of Oxford, Oxford, UK) (62). *Pax7-CreER^T2^* mice were provided by Cheng-Min Fan (Baltimore, Maryland, USA) (63). NCre nude mice were purchased from Taconic Biosciences. To minimize the effects of genetic background, littermate controls were used. Mice were randomly assigned into experimental treatment cohorts within cages to ensure balancing of sex and age. No mice used in this study received any treatment prior to their use in the experiments described in this paper.

#### Generation of isogenic cell lines and cell culture.

To generate *Atrx* isogenic mouse sarcoma cell lines, pSP Cas9(BB)-2A-GFP (PX458) with *Atrx* gRNA or vector control were transfected into newly derived primary soft tissue sarcoma cell lines (KP) using the TransIT-LT1 transfection reagent (Mirus). Cell lines from transfected single-cell clones were then screened for loss of ATRX protein expression by Western blot. Antibodies used for Western blot and immunofluorescence are listed in Supplemental Table 1. To generate human sarcoma cell lines, 143B cells were purchased (ATCC, CRL8303) and an identical procedure was performed, except the gRNA in the PX458 vector was targeted to the human *ATRX* gene. Passaging of sarcoma cell lines was performed in DMEM (Thermo Fisher Scientific) supplemented with 10% FBS (Thermo Fisher Scientific), 1% Pen-Strep (Thermo Fisher Scientific), and 1% L-glutamine (Thermo Fisher Scientific). Cell lines were incubated at 37°C with 5% CO_2_ in a humidified cell culture incubator. U2OS (HTB-96) and 143B (CRL-8303) cell lines were purchased from ATCC.

### Sarcoma induction

Primary sarcomas were generated in *Pax7^CreER^*; *LSL-Kras^G12D^*; *Trp53^Fl/Fl^* (P7 KP) and *Pax7^CreER^*; *LSL-Kras^G12D^*; *Trp53^Fl/Fl^*; *Atrx^Fl^* (P7 KPA) mice as previously described (64). To activate CreER in a spatially and temporally controlled manner in Pax7-expressing muscle satellite cells, 4-hydroxytamoxifen (4-OHT, Sigma-Aldrich) was dissolved in 100% DMSO at a concentration of 20 mg/mL and 25 μL of the 4-OHT solution was injected via intramuscular injection into the left mouse gastrocnemius muscle. For P7 P + MCA and P7 PA + MCA models, 4-OHT intramuscular injection was followed within 30 minutes by injection into the same muscle with 300 μg MCA (Sigma-Aldrich) resuspended in sesame oil (Sigma-Aldrich) at 6 μg/μL. Sarcoma cell lines for in vitro deletion of *Atrx* by CRISPR/Cas9 were generated in *LSL-Kras^G12D^*; *Trp53^Fl/Fl^* (KP) mice on a 129 Sv/Jae or mixed 129 Sv/Jae and C57/Bl6 background by injection of Cre-expressing adenovirus into the gastrocnemius muscle as previously described (24). KP *Atrx* WT and KP *Atrx* KO transplant sarcomas were generated by injecting 50,000 cells in a 1:1 mixture of DPBS (Gibco) and high-concentration matrigel (Corning) into the gastrocnemius muscle. Mice were anesthetized with 2% isoflurane prior to all injections or procedures. Human sarcoma xenografts were generated by injecting 1 million 143B cells with or without ATRX KO into Fox Chase SCID beige mice (Charles River) using Matrigel in the same manner as described above.

### Radiation therapy

When tumors reached 50–250 mm^3^ (Day 0, D0), mice were randomized to treatment groups, then tumor growth was monitored 3 times each week with caliper measurements in 2 dimensions**.** Sarcoma irradiation was performed using the Precision Xrad 225 Cx small animal image-guided irradiator (65). The radiation therapy field was centered on the mouse gastrocnemius tumor via fluoroscopy with a 40 kilovolt peak (kVp), 2.5 mA x-rays using a 0.3 mm copper filter. Sarcomas were irradiated with parallel- opposed anterior and posterior fields with an average dose rate of 300 cGy/minute prescribed to a midplane with 225 kVp, 13 mA x-rays using a 0.3-mm copper filter and a collimator with a 40×40 mm^2^ radiation field at treatment isocenter. Mice were euthanized using CO_2_ if they met their humane endpoint as determined by the IACUC protocol (moribund or in distress) or when tumor volume exceeded 2cm^3^, in accordance with Duke IACUC guidelines. Mice that were sacrificed for nontumor related reasons were excluded from analysis. Graphpad Prism version 9 was used for nonlinear fit modeling of tumor growth after irradiation.

### In vivo TVEC oncolytic herpesvirus treatment of P7 KPA and P7 KP mouse tumors

When tumors reached 50–250 mm^3^ (Day 0, D0), mice were randomized to treatment groups, then tumor growth was monitored 3 times each week with caliper measurements in 2 dimensions. A stock solution of a variant of TVEC, which differs from clinically approved TVEC in that it contains mouse GM-CSF instead of human GM-CSF, that had a concentration of 1×10^8^ PFU/mL was obtained and was diluted in DMEM at a ratio of 1 part TVEC stock solution to 2 parts DMEM. 50μl of this diluted TVEC solution was then injected into the central portion of the tumor mass. Mice were anesthetized with 2% isoflurane prior to all injections or procedures.

Further detailed methods are available in Supplemental methods and specific antibodies and reagent lists can be found in the supplemental key resources table. See complete unedited blots in the supplemental material.

### Statistics

For bar graphs, all data are presented as mean ± SEM. Two-tailed Student’s *t* tests with Welch’s correction) were used to compare the means of 2 groups unless analyzing results for multiple paired isogenic cell lines. For statistical analysis of multiple paired isogenic cell lines, a ratio paired 2-tailed *t* test was performed. Graphs with multiple comparisons were analyzed using a 2-way ANOVA with Tukey’s multiple comparisons test. For tumor growth experiments, survival curves were estimated for each group, considered separately, using the Kaplan-Meier method, and compared statistically using the log-rank test. A *P* value of less than 0.05 indicated significance. Prism 9 (GraphPad Software Inc.) was used for statistical analysis.

### Study approval

All animal studies were approved by the IACUC at Duke University.

### Data code and availability

RNA-Seq data has been submitted to Gene Expression Omnibus at NCBI, and is publicly available under GEO accession number GSE167537.

## Author contributions

WF and DGK conceptualized the project. WF, SBD, JAS, WCE, and DGK developed the methodology. WF, DLC, and DMC conducted the formal analysis. WF, MP, RP, CS, LL, ALL, KD, DZ, YM, and NTW performed the investigation. AJW, SBD, JAS, and WCE acquired resources for the study. WF and DGK wrote the original draft of the manuscript. WF, KD, AJW, DMC, and DGK reviewed and edited the manuscript. WF was responsible for visualization. DGK supervised the project.

## Supplementary Material

Supplemental data

Supplemental table 1

## Version 1 05/18/2023

In-Press Preview

## Version 2 07/03/2023

Electronic publication
