# Supplementary material for: Atrx deletion impairs CGAS/STING signaling and increases sarcoma response to radiation and oncolytic herpesvirus
Source: J Clin Invest. 2023 Jul 3;133(13):e149310. doi: 10.1172/JCI149310 (PMC10313374; doi:10.1172/JCI149310)
Supplement: Supplemental data [file jci-133-149310-s189.pdf]

Supplemental Methods

Immunohistochemistry

Tumors were fixed in 10% neutral buffered formalin overnight, transferred to 70% ethanol, paraffin- embedded, and sectioned to 5 μm thickness. Deparaffinized and rehydrated slides were blocked with 3% hydrogen peroxide. Antigen retrieval was performed by boiling slides in 2% citrate-based antigen unmasking solution (VECTOR H-3300) for 15 min. Slides were blocked with 5% normal serum in PBS + 0.25% Tween-20 and incubated overnight at 4C with primary antibodies as listed in Supplementary Table 1. Following this step, slides were diluted in PBS + 0.25% Tween-20 + 5% normal serum overnight at 4°C. After washing, slides were incubated with biotinylated secondary antibodies, including 1:200 horse anti-mouse IgG (VECTOR BA-2000) or 1 : 200 goat anti-rabbit IgG (VECTOR BA-1000) for 1 hour at room temperature. Slides were incubated with VECTASTAIN Elite ABC Reagent (VECTOR PK-7100) for 30 min at room temperature before signal was visualized with the 3,3′-diaminobenzidine (DAB) peroxidase substrate kit (VECTOR SK-4100). Representative images were acquired with a Leica DFC450 brightfield microscope using Leica Suite software (Leica Microsystems).

Hematoxylin and eosin (H&E) stained tissue sections were examined by a sarcoma pathologist (D.M.C.) blinded to genotype and treatment. For quantification of tumor necrosis, the sarcoma pathologist outlined areas of necrosis. ImageJ was used to quantify percentage of tissue that was identified as necrotic. Tumor type was classified by D.M.C. based on the full panel of sarcoma classification markers that utilized 9 antibodies (Supplementary Table 1). For Ki-67 IHC experiments, ImageJ was used to quantify positively stained cells by a single blinded observer.

in vitro Immunocytochemistry

Nunc Lab-Tek II Chambered Coverglass was used to grow and image cells (ThermoFischer

Scientific, 155379). Cells were fixed with 4% paraformaldehyde, blocked and washed using the MAXpack Immunostaining Media Kit (Active Motif), then incubated with primary antibody as listed in Supplementary Table 1 overnight at 4°C. Secondary antibody (Supplementary Table 1) was incubated for 1 hour at room temperature. Cells were stained with DAPI and then Prolong Diamond Antifade Mountant. Imaging was performed on a Leica SP5 Inverted Confocal Microscope, with consistent settings to allow for comparison. For each experiment a minimum of 50 cells were imaged then analyzed by a blinded researcher using Leica Suite software (Leica Microsystems). Data from these experiments represent the average of at least three independent experimental technical replicates.

TUNEL Assay

Frozen tissue sections were allowed to thaw at room temperature for 5 minutes, then sections were fixed in cold 4% paraformaldehyde for 10 minutes. Samples were washed twice with ice cold PBS, then incubated with 0.1% Triton X-100 and 0.1% sodium citrate in water for five minutes. Samples were washed 3 times for 5 minutes each in PBS. Then *in situ* Cell Death Detection Kit, *AP* (Roche, #11684809910) was used. 100 microliter TUNEL reaction mixture from this kit was added to each sample and incubated for 1 hour at 37°C. Each sample was washed three times with PBS, mounted, and then imaged with a DFC340FX fluorescence microscope using Leica Software.

Mitotic Dysfunction Assays

Micronuclei were defined as regions of DAPI staining located in close proximity to a nucleus with a size between approximately 1/3 and 1/64 the size of a nucleus, which could be determined to be distinct from the nucleus. Chromosome bridges were defined as occurrences of a single thin clear line

connecting two otherwise separate nuclei. For telomere dysfunction induced foci (TIF), which are 53BP1 foci that co-localized with telomeres, co-localization was defined by visual inspection in multiple channels and were counted if there was an instance of a 53BP1 focus that was contiguous or overlapping with a telomere focus. Only a single TIF event was called per 53BP1 focus, even if multiple telomere foci were present. Mitotic catastrophe was identified by the presence of multiple micronuclei that were associated with a single nucleus (1-3).

ImmunoFISH

Telomere ImmunoFISH was performed as described in (4) (Basic Protocol 1), with the following modifications: Nunc Lab-Tek II Chambered Coverglass was used to grow and image cells (ThermoFischer Scientific, 155379). Cells were incubated for 30 seconds in pre-extraction buffer, and Alexa-Fluor 488 conjugated telG FISH probe was used (PNA BIO). For the FISH hybridization step, slides were preheated for 1 minute before adding the telomere probe to minimize background staining. Imaging was performed on a Leica SP5 Inverted Confocal Microscope, and imaging for each experiment was performed using consistent settings to allow for comparison.

Cell Line Generation from Primary Sarcomas

Tumors were collected immediately after mouse sacrifice. Tumor tissue was minced and digested in dissociation buffer in PBS (Thermo Fisher Scientific, 14040133) containing collagenase type IV (5 mg/ml, Thermo Fisher Scientific, 17104-019), dispase (1.3 mg/ml, Thermo Fisher Scientific, 17105-041) as well as trypsin (0.05%, Thermo Fisher Scientific, 25200056) for about 1 h at 37°C in a cell culture hood and incubator. Cells were washed with PBS (Thermo Fisher Scientific, 10010023) and filtered using a 40 μm sieve (Corning, 431750) and cultured for at least five passages to deplete stroma before being used for experiments. To assess

recombination of the *Atrx* floxed allele, tumor cell line genomic DNA was isolated and genotyped by PCR to amplify exon 18 or the recombined *Atrx*^{FL} allele using primer sets in Supplementary Table 1.

IC50 assays

For IC50 assays with doxorubicin, cells were plated at 1000 cells per well in a 96 well plate, then treated with 1:2 serial dilutions of doxorubicin. Cells were incubated in a cell culture incubator with 5% carbon dioxide at 37 degrees Celsius for 72 hours before cells were washed twice with PBS then measured via CellTiter-Glo (Promega). IC50 experiments were repeated three times for each cell line shown. For IC50 assays with Imanis oHSV, cells were plated at 1000 cells per well in a 96 well plate, then treated with 1:3 serial dilutions of Imanis oHSV (starting stock concentration 4.4x10^7 PFU/mL). Plates were incubated for 72 hours and then measured via CellTiter-Glo as above. For IC50 assays with TVEC, a stock solution of a variant of TVEC (this variant differs from clinically approved TVEC in that it contains mouse GM-CSF instead of the human GM-CSF) that had a concentration of 1X10^8 PFU/ml was obtained and diluted 1:10 in DMEM. ATRX isogenic 143B human sarcoma cells were plated at 1000 cells per well in a 96 well plate, then treated with 1:2 serial dilutions of the prepared solution. Cells were incubated in a cell culture incubator with 5% CO_2 at 37°C for 72 hours before cells were washed twice with PBS then measured via CellTiter-Glo (Promega). IC50 experiments were repeated three times for each cell line shown.

Genotyping

Mice were genotyped using tails collected from mouse pups. Tail genomic DNA was extracted using a KAPA mouse genotyping Kit (KAPA Biosystems KK7352), and PCR was performed using primers as described in Supplementary Table 1. Additional genotyping was performed by

shipping mouse tail snips to Transnetyx, where DNA was extracted and analyzed using a Taqman qpcr assay. For *Atrx* exon 18 assay (Figure 2E, top), presence of un-recombined exon 18 is shown by the presence of a band at 1000 bp, while lanes with no band present indicates successful recombination. For *Atrx* recombination PCR assay (Figure 2E, bottom) , *Atrx* WT can be visualized as a 1400 bp band, floxed *Atrx* allele as a 1600 bp band, and the recombined *Atrx* allele as a 600bp band.

Immunoblotting

Samples were lysed in RIPA buffer for 30 min on ice (Sigma-Aldrich, R0278), sonicated briefly, and then centrifuged at 10,000g for 10 min at 4°C. Protein concentration was determined for the lysate supernatant by BCA assay (Pierce, 23225). Samples were boiled in 4x Laemmli sample buffer (Bio-Rad, 1610747) before loading in a 4–20% Tris-glycine polyacrylamide gel. Membranes were blocked in 1:1 TBS:Intercept blocking buffer (LiCor) for 1 hour. Next, the membranes were incubated overnight at 4°C with primary antibodies diluted in TBS-T (0.1% Tween-20) at concentrations as indicated in Supplementary Table 1. The membranes were washed three times in TBS-T before secondary antibody incubation with goat anti-rabbit IRDye800 (Li-Cor Biosciences, P/N 925-32211) and goat anti-mouse IRDye680 (Li-Cor Biosciences, P/N 925-68070) both at 1:10,000 dilutions in TBS-T for 1h at room temperature. The membranes were washed three times in TBS-T and imaged using an Odyssey CLx (Li- Cor Biosciences). Image analysis for normalization and quantification was performed using Image Studio (version 5.2, Li-Cor Biosciences, P/N 9140-500).

rt-PCR

Cells were lysed with TRIzol reagent (Thermo Fisher, 15596026). RNA was isolated from samples

using a Direct-zol RNA MiniPrep kit (Zymo Research, R2051). RNA samples were reverse transcribed to cDNA using an iScript Advanced cDNA Synthesis Kit (Bio-Rad, 1725038). TaqMan probes from Thermo Fisher were used for RT-PCR: mouse probes were *IfnB1* (Mm00439552), *Gapdh* (Mm99999915), *Tbp* (Mm01277042_m1) Ifit1 (Mm07295796), Cxcl10 (Mm00445235), Ifit3 (Mm00445235), and Rsad2 (Mm00491265). Human probes were IFNB1 (Hs01077958) and ACTB(Hs01060665). Taqman fast advanced master mix was used for the qPCR assay (ThermoFischer Scientific 4444556). Plates were run on a QuantStudio 6 Flex Real-Time PCR System (Thermo Fisher), and data were analyzed using the comparative C_T method to generate expression fold-change values. *Gapdh* or *Tbp* expression was used as an internal control for RNA concentration across samples. Every sample was run in triplicate and results were averaged for each assay.

Mouse Whole Exome Sequencing

Mouse whole exome sequencing was performed by Novogene using their standard protocol for sample preparation and data analysis. Each mouse tumor was submitted along with a portion of normal muscle from the same mouse, then WES was performed by Novogene. Briefly The genomic DNA was randomly sheared into short fragments with the size of 180-280 bp. The obtained fragments were end repaired, A-tailed, and further ligated with Illumina adapters. The fragments with adapters were PCR amplified, size selected, and purified. Hybridization capture of libraries was proceeded according to the following procedures. Briefly, the prepped libraries were hybridized in the buffer with biotin-labeled probes, and magnetic beads with streptavidin were used to capture the exons of genes. Subsequently, non-hybridized fragments were washed out and probes were digested. The captured libraries were enriched by PCR amplification. The original fluorescence images obtained from high throughput sequencing platforms were

transformed to short reads by base calling. These short reads (Raw data) are recorded in FASTQ format, which contains sequence information (reads) and corresponding sequencing quality information.For quality control, read pairs were discarded if either one read contained adapter contamination, if more than 10% of bases in either read were uncertain, or if the the proportion of low quality bases was over 50% in one read. Q30 was required to above 80% for the samples. Burrows-Wheeler Aligner (BWA) was utilized to map the paired-end clean reads to the mouse reference genome (mm10). After sorting with Samtools and marking duplicates with Picard, the results of read alignment was finally stored in the format of BAM. The coverage and depth was then computed based on the final BAM file. Somatic SNP/InDel detection and filtering was performed using GATK, as well as MuTect/Strelka. Somatic CNV detection was performed using Control-FREEC, and annotation was performed using ANNOVAR.

RNA-seq Bioinformatic Analysis

Raw counts from Novogene's processing pipeline were obtained from the company. For cell line RNA seq analysis, genes were first filtered if they did not have at least 10 reads in a single sample from each of the 3 replicates. Normalization and differential expression were carried out using the DESeq2 Bioconductor package with the R statistical programming environment. In each analysis, 'replicate' was used as a cofactor in the model. The false discovery rate was calculated to control for multiple hypothesis testing. Gene set enrichment analysis (5) was performed to identify gene ontology terms and pathways associated with altered gene expression for each of the comparisons performed.

Cancer Genomic Database Analysis

For determination of the frequency of ATRX mutation in human cancers, the TCGA database was queried via cBioportal. For overall prevalence of ATRX in human cancer, "Curated set of non-redundant studies" was selected, and was queried for genomic profile of mutations and copy number alterations in ATRX. For data on frequency and position of ATRX mutations in soft tissue sarcoma, "Sarcoma (TCGA, PanCancer Atlas)" study was selected, UPS, LMS and MFS sarcoma subtypes were selected, then were queried for genomic profile of mutations and copy number alterations in ATRX. Disease free survival data was obtained via CBioportal. Whole genome sequencing mutation and phenotype data of undifferentiated pleomorphic sarcomas was accessed from (2). Samples harboring frameshift or missense mutations in ATRX exons, as well as samples with copy number deletion, fusions or inversions were classified as "ATRX altered".

cGAMP ELISA Assay

The ELISA assay was performed for cGAMP according to the protocol provided (Cayman Chemical no. 501700). Briefly, 96-well plates were washed, ELISA standards and samples were added, and 2'3'-cGAMP-HRP Tracer and then 2'3'-cGAMP Polyclonal Antiserum were added to the wells. Plates were incubated overnight at 4C, HRP Stop Solution was added, and plate was read at 450 nm (Molecular Devices SpectraMax i3).

Cell Growth Assay

Mouse sarcoma primary isogenic cell lines were cultured in 96-well plates. After 60 hours, plates were equilibrated at room temperature for 30 minutes before addition of 100μl Cell Titer Glo Reagent (Promega CellTiter-Glo Luminescent Cell Viability Assay, 77573).

Reagents were mixed on a shaker for 2 minutes and incubated for 10 minutes at room temperature. Relative fluorescence was recorded using a luminometer (Molecular Devices

SpectraMax i3).

Supplemental Methods References:

1. Eriksson D, Lofroth PO, Johansson L, Riklund KA, and Stigbrand T. Cell cycle disturbances and mitotic catastrophes in HeLa Hep2 cells following 2.5 to 10 Gy of ionizing radiation. *Clin Cancer Res.* 2007;13(18 Pt 2):5501s-8s.
2. Vakifahmetoglu H, Olsson M, and Zhivotovsky B. Death through a tragedy: mitotic catastrophe. *Cell Death Differ.* 2008;15(7):1153-62.
3. Roninson IB, Broude EV, and Chang BD. If not apoptosis, then what? Treatment-induced senescence and mitotic catastrophe in tumor cells. *Drug Resist Updat.* 2001;4(5):303-13.
4. Cesare AJ, Heaphy CM, and O'Sullivan RJ. Visualization of Telomere Integrity and Function In Vitro and In Vivo Using Immunofluorescence Techniques. *Curr Protoc Cytom.* 2015;73:12 40 1-12 40 31.
5. Mootha VK, Lindgren CM, Eriksson KF, Subramanian A, Sihag S, Lehar J, et al. PGC-1alpha-responsive genes involved in oxidative phosphorylation are coordinately downregulated in human diabetes. *Nat Genet.* 2003;34(3):267-73.

Supplementary Figure 1

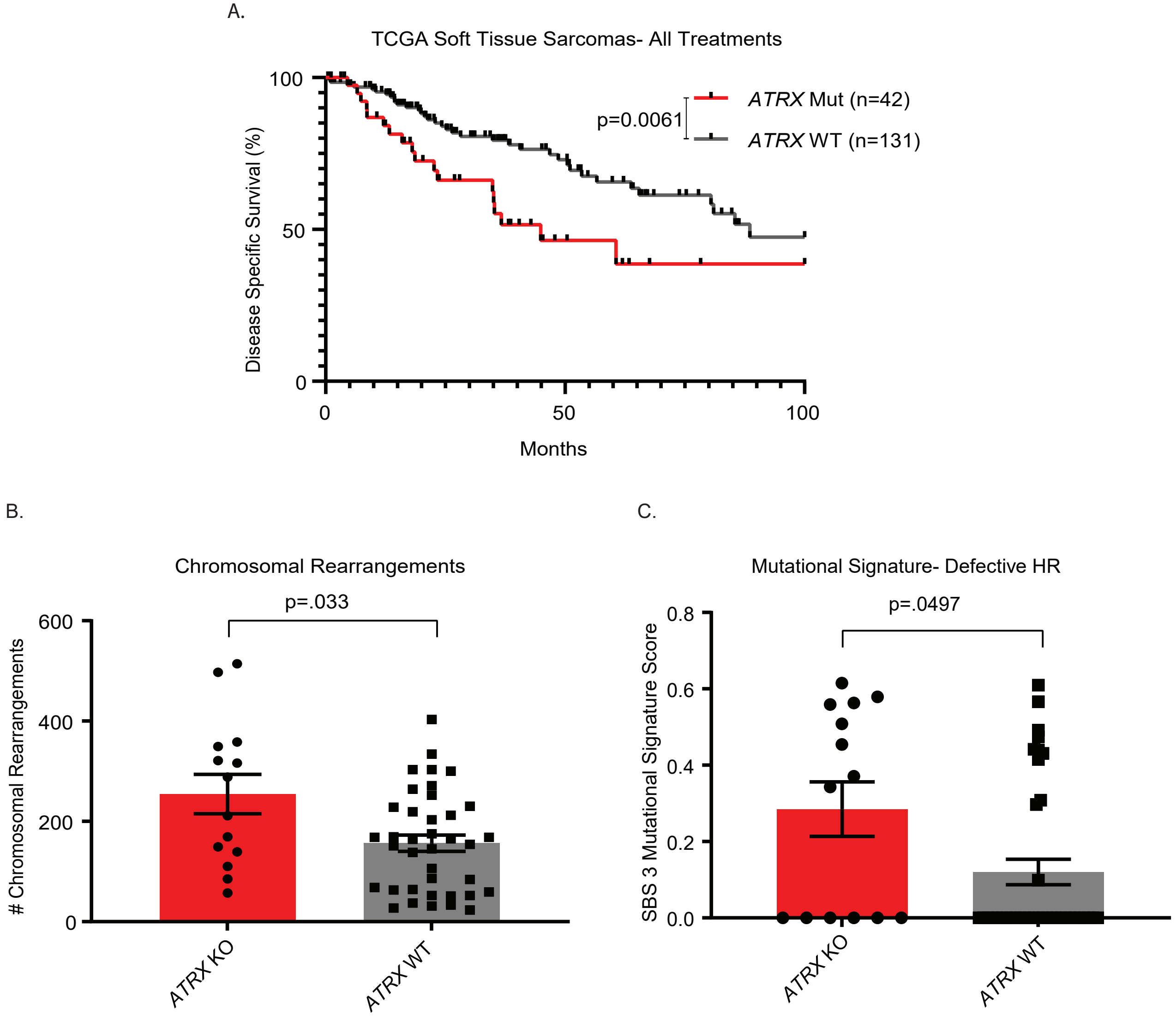

Supplementary Figure 1: Characteristics of Human Soft Tissue Sarcomas
A) Disease specific survival for a cohort of soft tissue sarcomas (leiomyosarcomas, myxofibrosarcomas, and undifferentiated pleomorphic sarcomas), comparing sarcomas with *ATRX* genomic alteration (*ATRX* Mut, n=42) to sarcomas without *ATRX* genomic alteration (*ATRX* WT n=131). Statistical comparison was performed using a log-rank (Mantel Cox) test. B) Analysis of whole genome sequencing of undifferentiated pleomorphic sarcoma showing average number of chromosomal rearrangements in 14 *ATRX* altered and 38 *ATRX* wild type (WT) human tumors. C) Analysis of whole genome sequencing of undifferentiated pleomorphic sarcoma showing COSMIC mutation signature 3 score, which reflects defective homologous recombination (HR)-based double strand DNA break repair, in 14 *ATRX* altered and 38 *ATRX* WT human tumors. Statistical analysis for B) and C) was performed using a Welch's t-test. Each data point represents a biological replicate.

Supplementary Figure 2

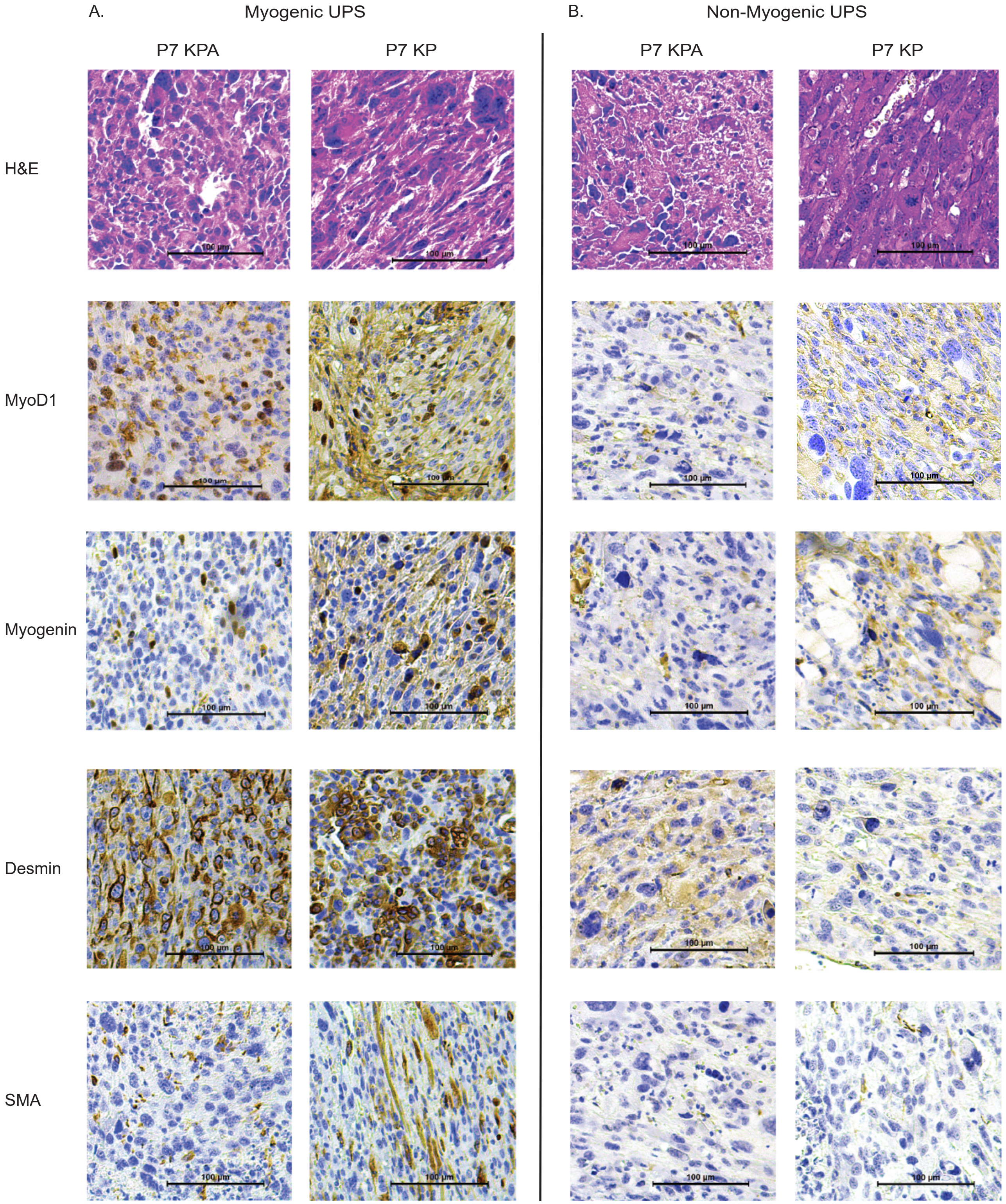

Supplementary Figure 2: Immunohistochemistry for classification of soft tissue sarcomas
A-B) Representative H&E staining and immunohistochemistry for myogenic markers for formalin fixed, paraffin embedded tumors used to classify soft tissue sarcomas from the P7 KPA (left) and P7 KP (right) mice. Myogenic UPS H&E staining images are identical to images shown in figure 2. H&E staining as well as immunohistochemistry of muscle markers MyoD (nuclear), Myogenin (nuclear), Desmin (cytoplasmic) and SMA (cytoplasmic) for myogenic UPS (A) and non-myogenic UPS (B). Tumors shown were classified as based on the full panel of sarcoma classification markers that utilized 9 antibodies (Supplementary Table 1). Scale bar is 100 um. H&E images shown at top of Supplementary Figure 2A also shown in main text Figure 2B

Supplementary Figure 3

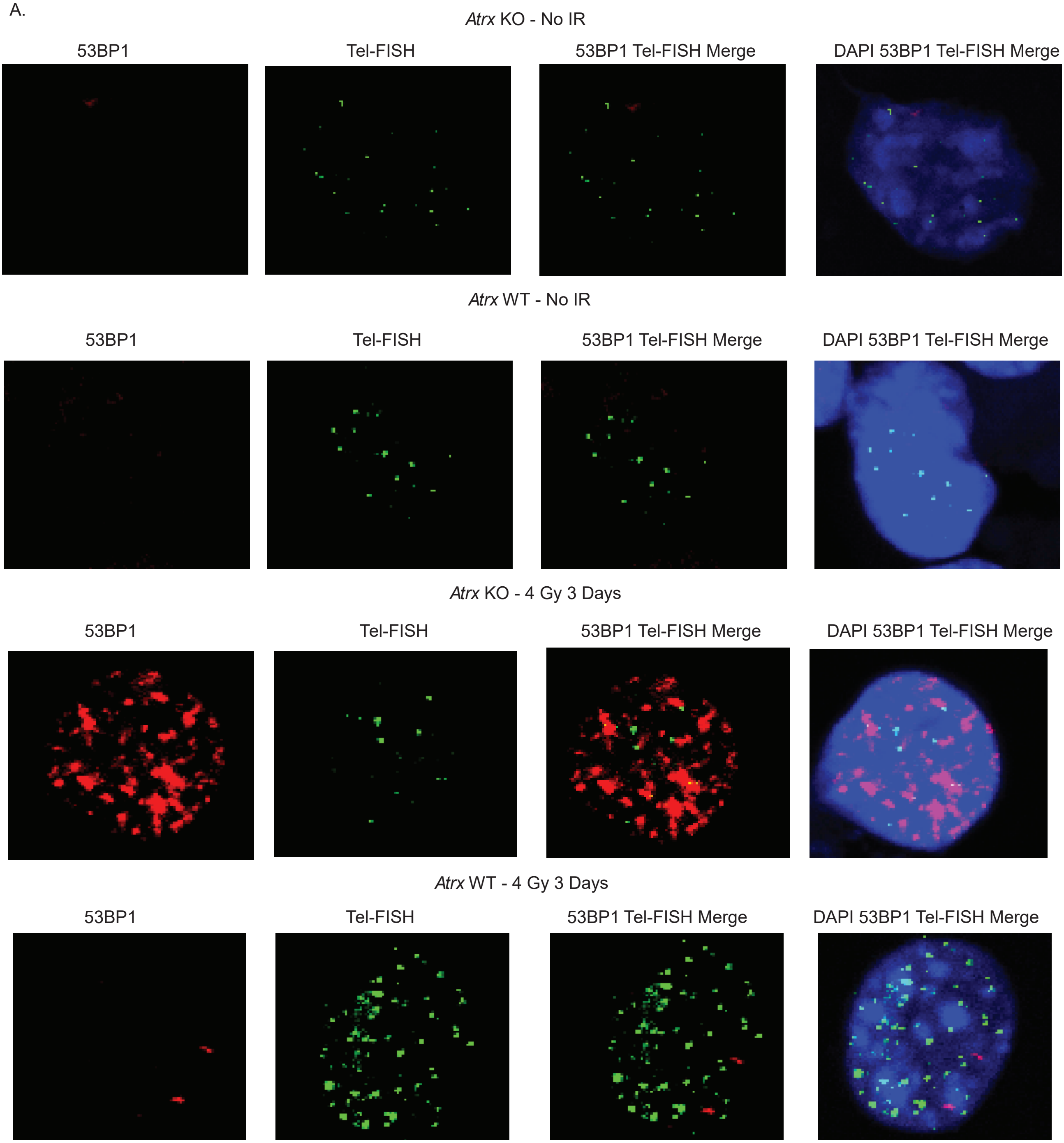

Supplementary Figure 3: ImmunoFISH staining
Representative images showing immunofish staining of an *Atrx* KO and WT isogenic cell line pair. Cells were fixed and stained 3 days after 4 Gy irradiation or no irradiation. Each series shows 53BP1 staining (red), telomere FISH staining (green), merged image of 53BP1 and telomere fish staining, and merge of 53BP1, telomere FISH, and DAPI staining.

Supplementary Figure 4

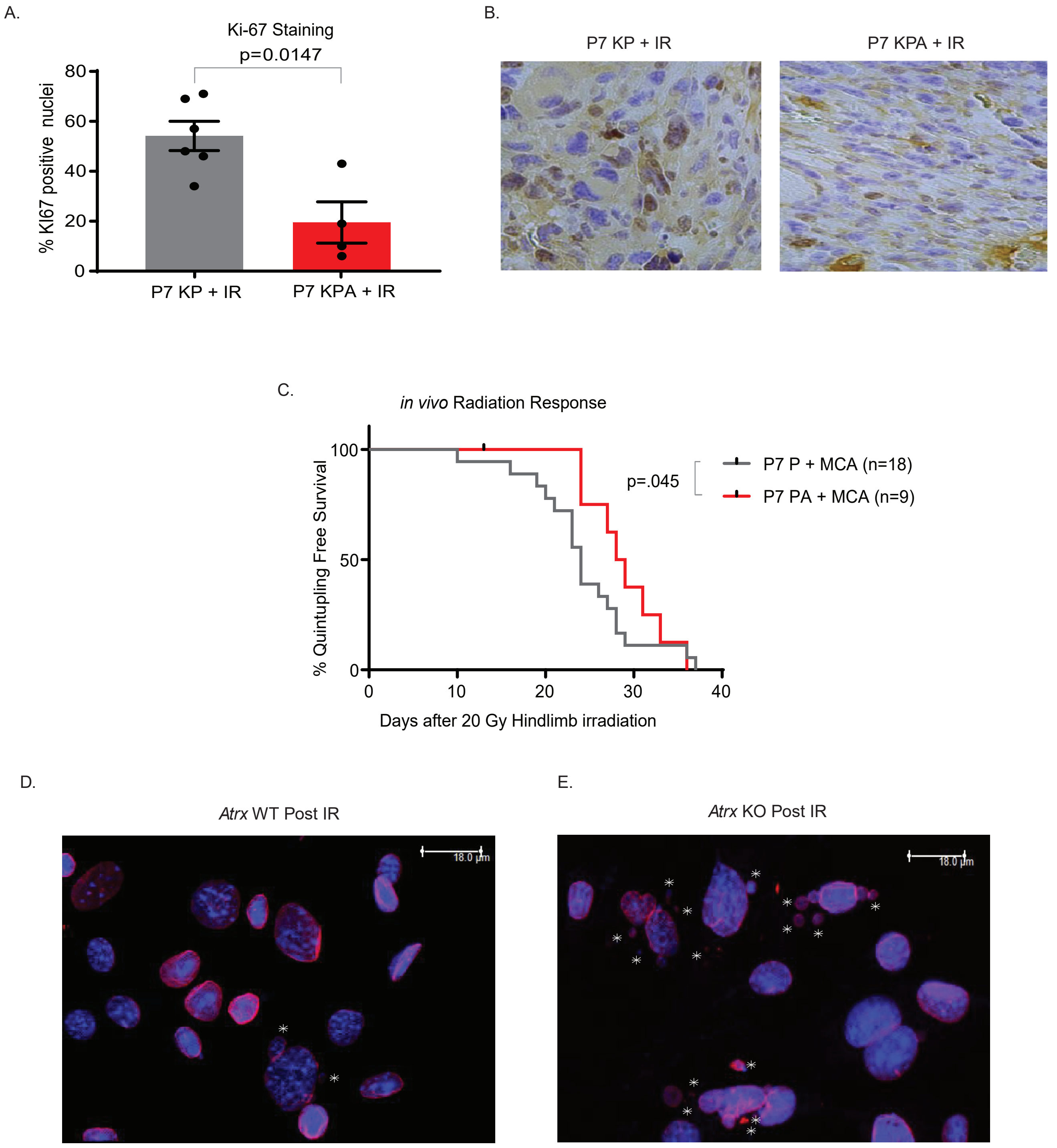

Supplementary Figure 4: Effect of *Atrx* deletion on radiation response in soft tissue sarcomas
A) Quantification of Ki67 staining from sarcomas from P7 KP (n=6) and P7 KPA (n=4) mice harvested 6 Days after 20 Gy. Each data point represents a biological replicate. Statistical comparison was performed using an unpaired t-test with Welch's correction B) Representative Ki67 staining from sarcomas from P7 KP (left) and P7 KPA (right) mice harvested 6 days after 20 Gy. C) Time to tumor quintupling after treatment with 20 Gy in the P7 P + MCA (n=18) and P7 PA+ MCA (n=9) primary mouse models of soft tissue sarcoma. Comparison of survival curves was performed using log-rank (Mantel Cox) test. D-E) Representative images of micronuclei in *Atrx* WT (D) and *Atrx* KO (E) sarcoma cells three days after 4 Gy as quantified in Figure 6A. DAPI is blue and Lamin B1 is red. The presence of nuclear envelope enclosed micronuclei can be observed (white stars).

Supplementary Figure 5

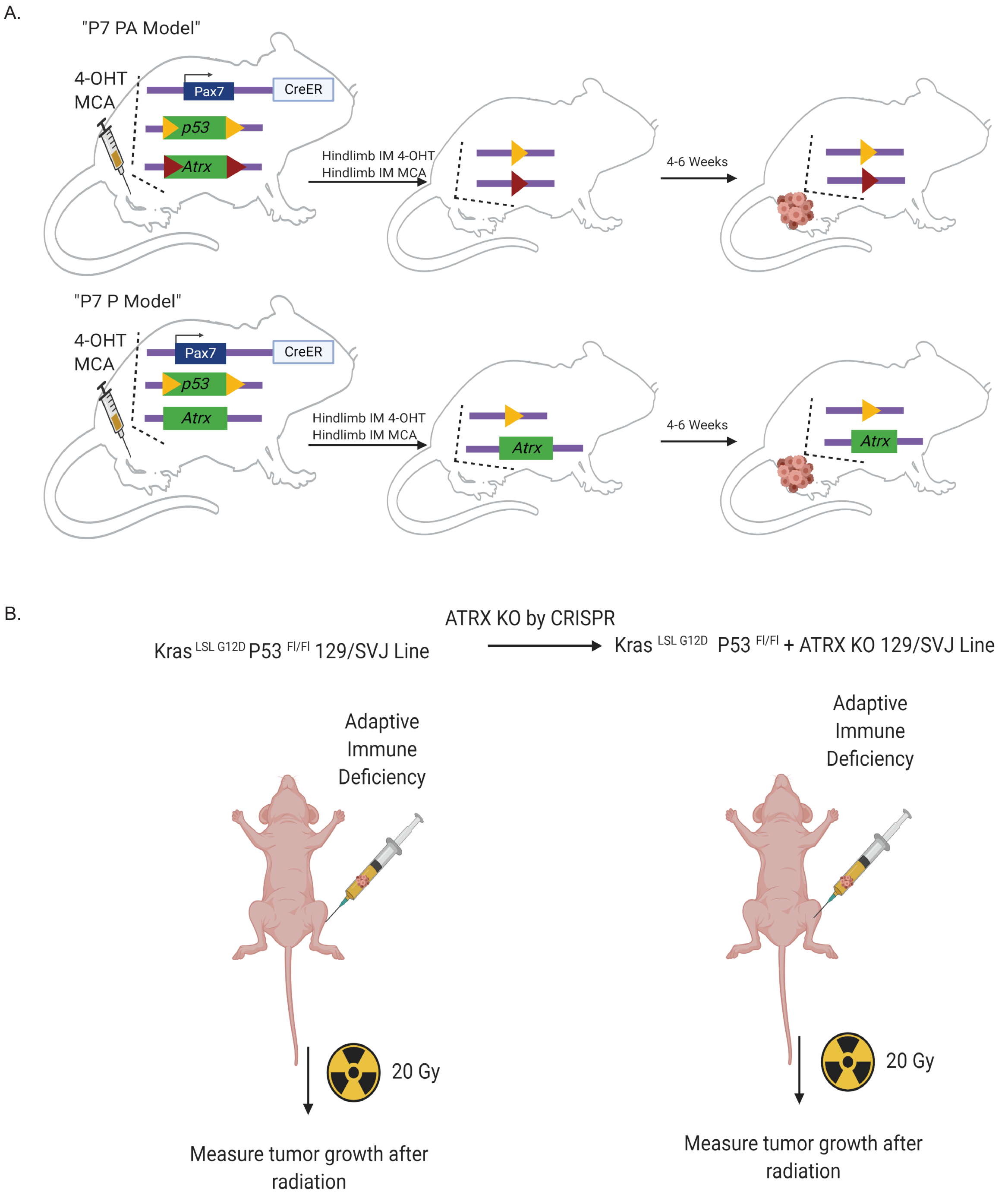

Supplementary Figure 5: Experimental schematics for mouse experiments
A) Diagram showing the P7 PA model of soft tissue sarcoma, triangles represent loxp sites. Mice receive intramuscular injection of 4-OHT in DMSO followed within 30 minutes by injection of MCA into the same muscle. The P7 P + MCA model does not utilize a genetically engineered conditional oncogenic $\textit{Kras}^{G12D}$ allele, but instead is initiated by intramuscular injection of 4-OHT in *Pax7-CreERT2*; $\textit{Trp53}^{fl/fl}$ mice to delete both *Trp53* alleles followed by injection of 3-methylcholan-threne, a potent carcinogen that drives base substitutions. The P7 PA + MCA model is identical except for the presence of loxp sites flanking exon 18 of *Atrx* that allows deletion of both *Atrx* and *Trp53* following administration of 4-OHT. B) Diagram showing the design of tumor transplant experiment into immunodeficient mice. Nude mice received hindlimb intramuscular injection of the gastrocnemius with 50,000 cells in Matrigel. After tumors were detected by caliper, mice were treated with 20 Gy and measured three times a week until tumors reached 2000 mm^3 or euthanasia was required by IACUC humane endpoints.

Supplementary Figure 6

A.

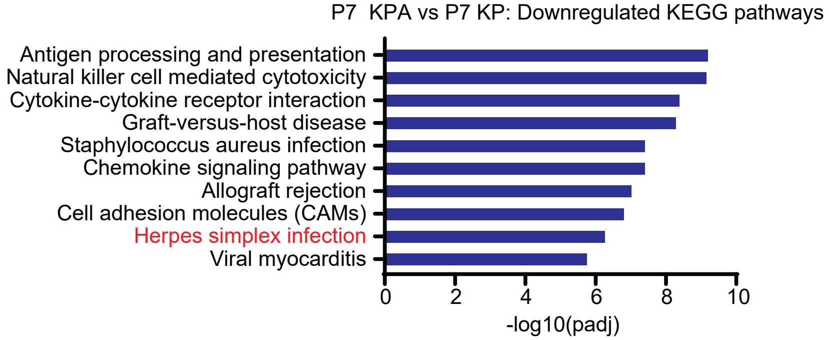

B.

Gene Name	P7KPA vs P7KP Log2FoldChange	P7KPA vs P7KP padj	In vitro ATRX KO vs WT log2FoldChange	In vitro ATRX KO vs WT padj
Tmem173	-0.533559842	0.820254252	-0.71434767	0.999985597
Mb21d1	-0.522242792	0.935390654	-0.744479346	0.999985597
Irf3	-0.801739708	0.624427893	-0.191160163	0.999985597
Tbk1	-0.12702029	0.987615604	0.105007437	0.999985597
Chuk	0.15302986	0.987615604	0.271369913	0.999985597
Ikbkb	-0.238465522	0.971127727	-0.351745955	0.999985597
Rela	-0.040765671	0.997352858	0.138547488	0.999985597
Relb	-0.431769879	0.882435962	-0.135715795	0.999985597
Atm	0.288790004	0.987615604	-0.192069394	0.999985597

C.

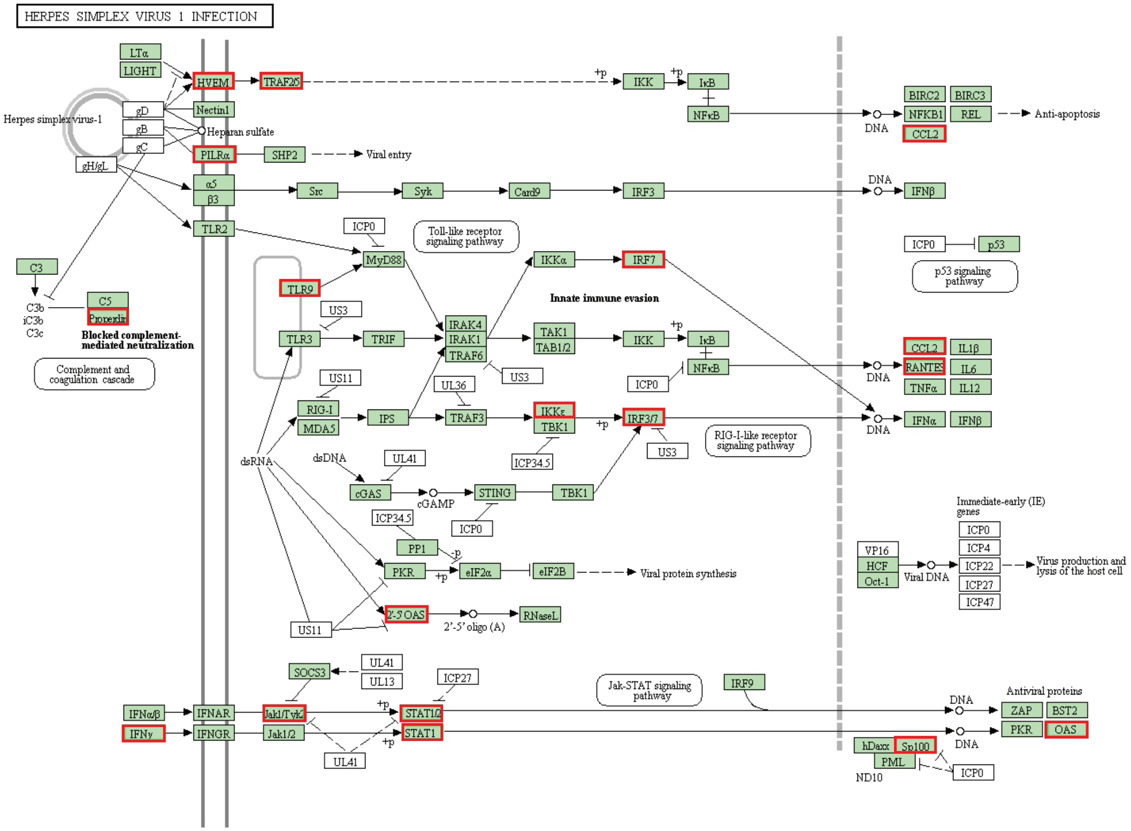

Supplementary Figure 6: *ATRX* deletion results in reduced expression of HSV-1 innate immune defense pathways
A) Top ten results for KEGG enrichment analysis of RNA sequencing downregulated pathways comparing unirradiated P7 KPA sarcomas (n=4) to unirradiated P7 KP tumors (n=4). HSV pathway enrichment highlighted in red text. B) RNA sequencing and analysis of expression levels of CGAS and STING related pathway genes in untreated *Atrx* WT and *Atrx* deleted in vitro and in vivo models. C) Partial KEGG pathway map for cellular response to HSV-1 infection. Cellular genes are in green, HSV genes are in white. Red boxes are used to indicate genes that are downregulated (P7 KPA No IR vs P7 KP No IR) in the KEGG pathway, no genes were upregulated in this KEGG pathway.

Supplemental Figure 7

A.

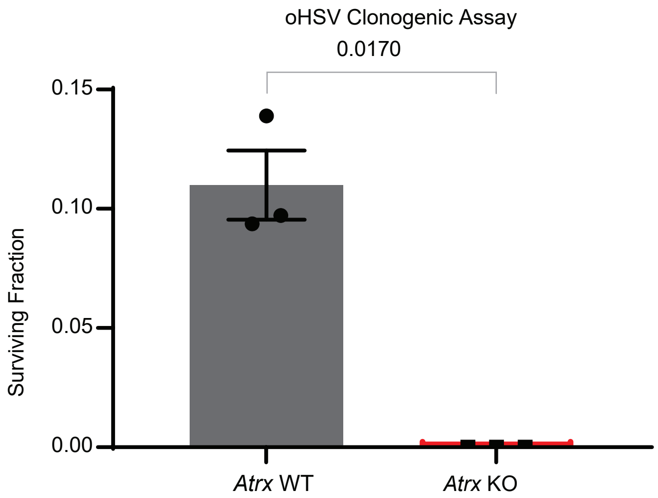

B.

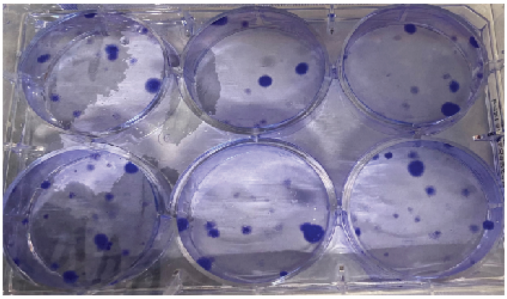

Atrx WT - oHSV

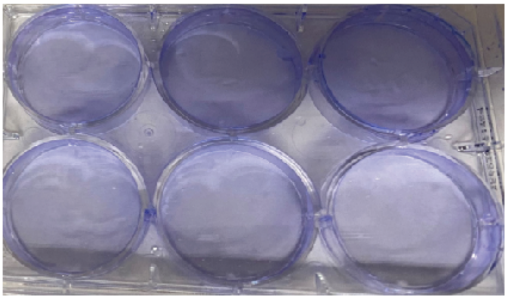

Atrx KO - oHSV

Supplementary Figure 7: Oncolytic Herpesvirus Therapeutic Effect
A) Quantification of colonies in *Atrx* retained (n=3) and *Atrx* deleted (n=3) isogenic cell line pair after oncolytic herpesvirus (Imanis oHSV) treatment. Each dot represents an experimental repeat using the same cell line pair. Statistical comparison was performed using an unpaired t-test with Welch's correction. B) *Atrx* WT and *Atrx* KO paired isogenic cell lines were plated in triplicate at identical density and treated with Imanis oHSV at a concentration corresponding to the IC90 of the *Atrx* WT cell line. Colonies were stained with crystal violet.

Supplementary Figure 8

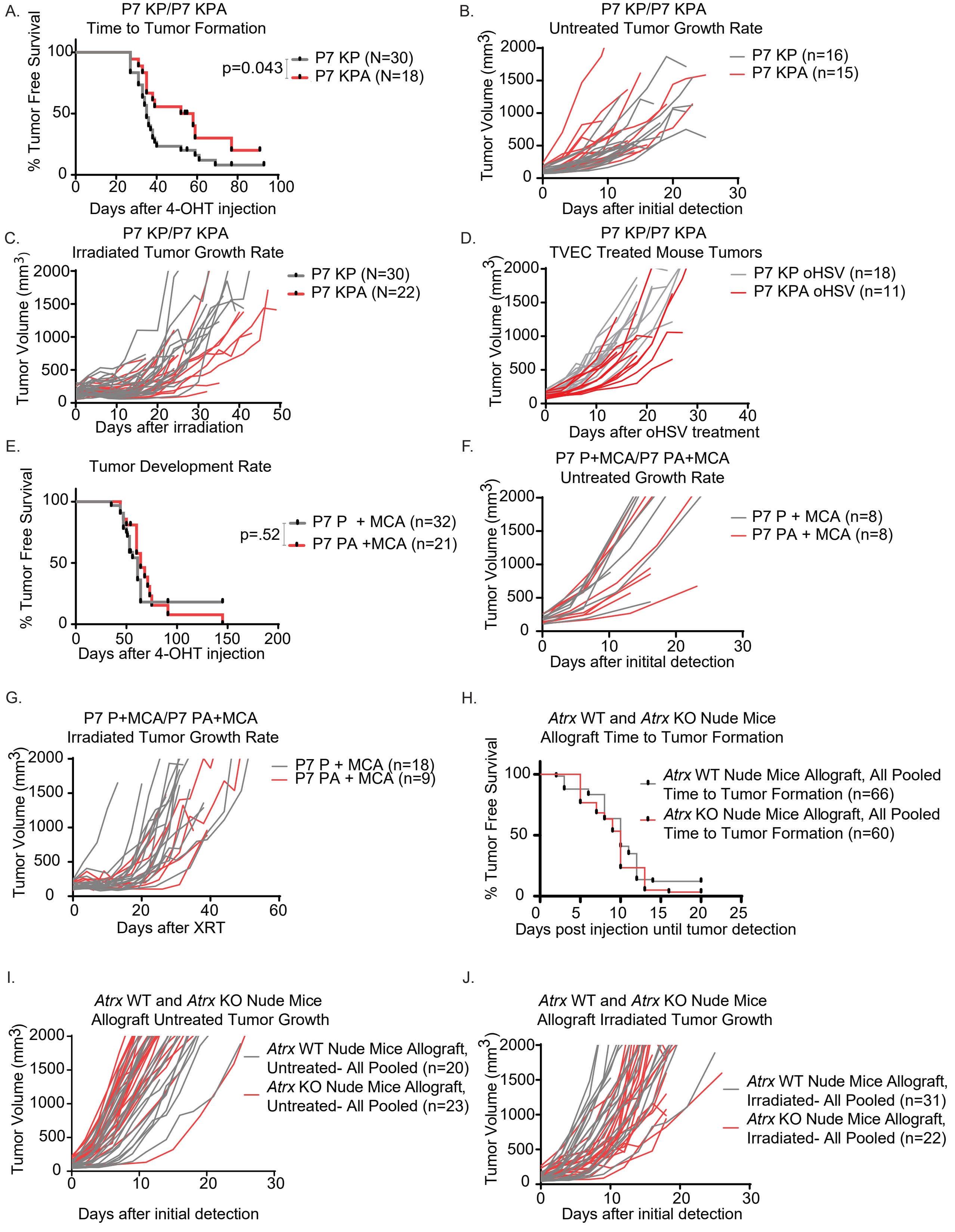

Supplementary Figure 8 continued

Supplementary Figure 8: In Vivo Sarcoma Growth Kinetics
A) Time from tumor initiation to tumor detection by palpation, as measured by tumor free survival for sarcomas in P7 KP (n=30) and P7 KPA (n=18) mice. Comparison of curves was performed using log-rank (Mantel Cox) test. B-D) Individual growth curves for untreated (B), Irradiated (C) or TVEC oHSV treated (D) sarcomas in P7 KP and P7 KPA mice. Number of mice shown in legend for each graph. Each line in these graphs represents an individual tumor’s growth over time. E) Time from tumor initiation to tumor detection by palpation, as measured by tumor free survival for P7 P+ MCA (n=32) and P7 PA + MCA (n=21) tumors. Comparison of survival curves was performed using log-rank (Mantel Cox) test. F-G) Individual growth curves for untreated (F) and irradiated (G) sarcomas in P7 P+MCA and P7 PA+MCA models. Number of mice shown in legend for each graph. Each line in these graphs represents an individual tumor’s growth rate over time. H) Time from tumor cell line injection in nude mice to tumor detection by palpation, as measured by tumor free survival for xenograft with 143B human sarcoma cell lines with (n=66) or without *Atrx (n=60)*. I-J) Individual growth curves for untreated (I) and irradiated (J) nude mice xenografted with 143B human sarcoma cell lines with or without *ATRX*. Number of mice shown in legend for each graph. Each line in these graphs represents an individual tumor’s growth rate over time.

Supplementary Figure 9

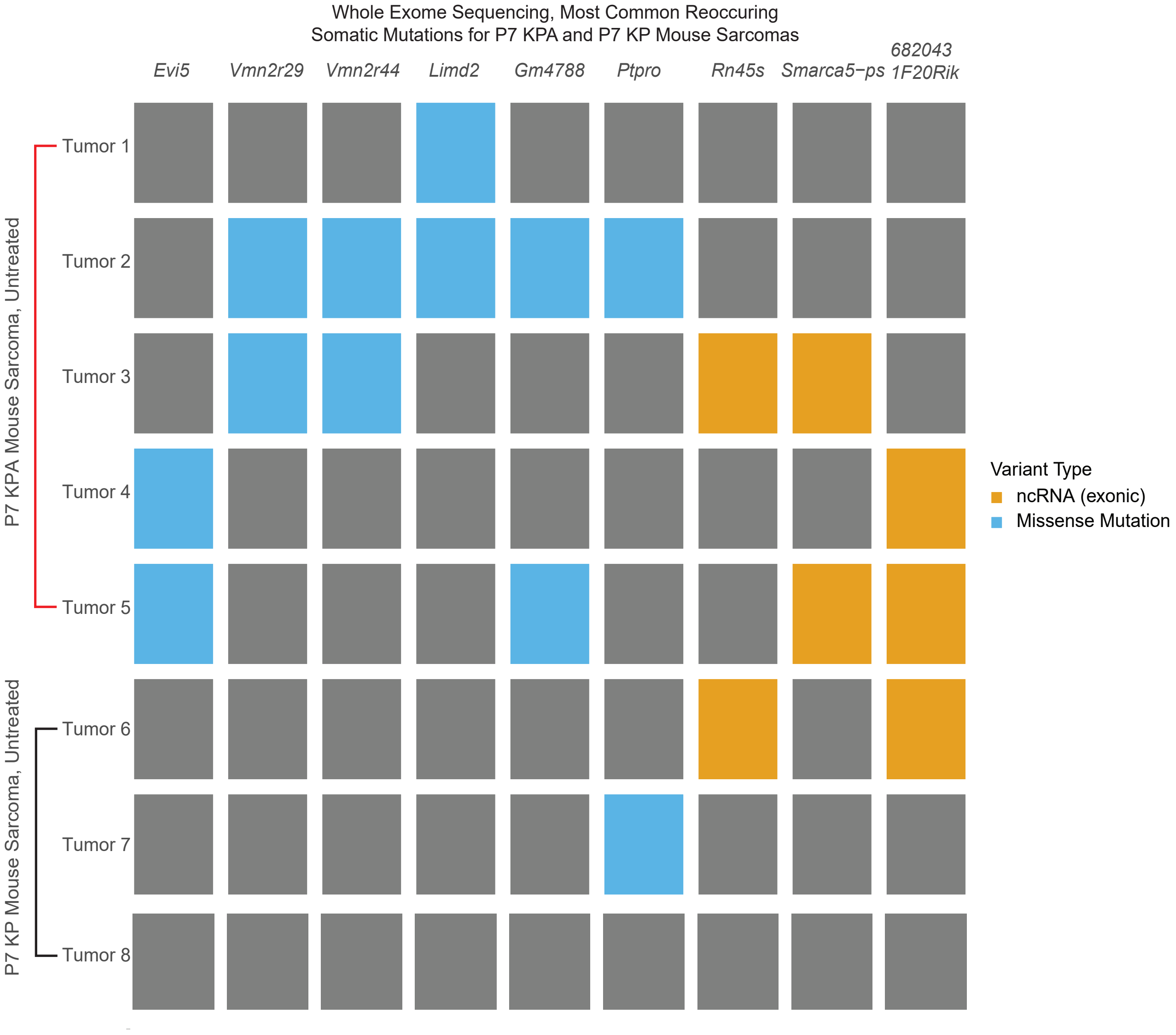

Supplementary Figure 9: Whole Exome Sequencing of Most Frequent Genomic Alterations in Mouse Sarcoma With Deleted *Atrx*
Table showing most frequent recurrent mutations in sarcomas from P7 KPA and P7 KP mice. Each row is an individual tumor sequenced with mutations identified when compared to its matched normal tissue. Columns represent genes that had the highest number of tumor samples with mutations.

Supplemental Figure 10

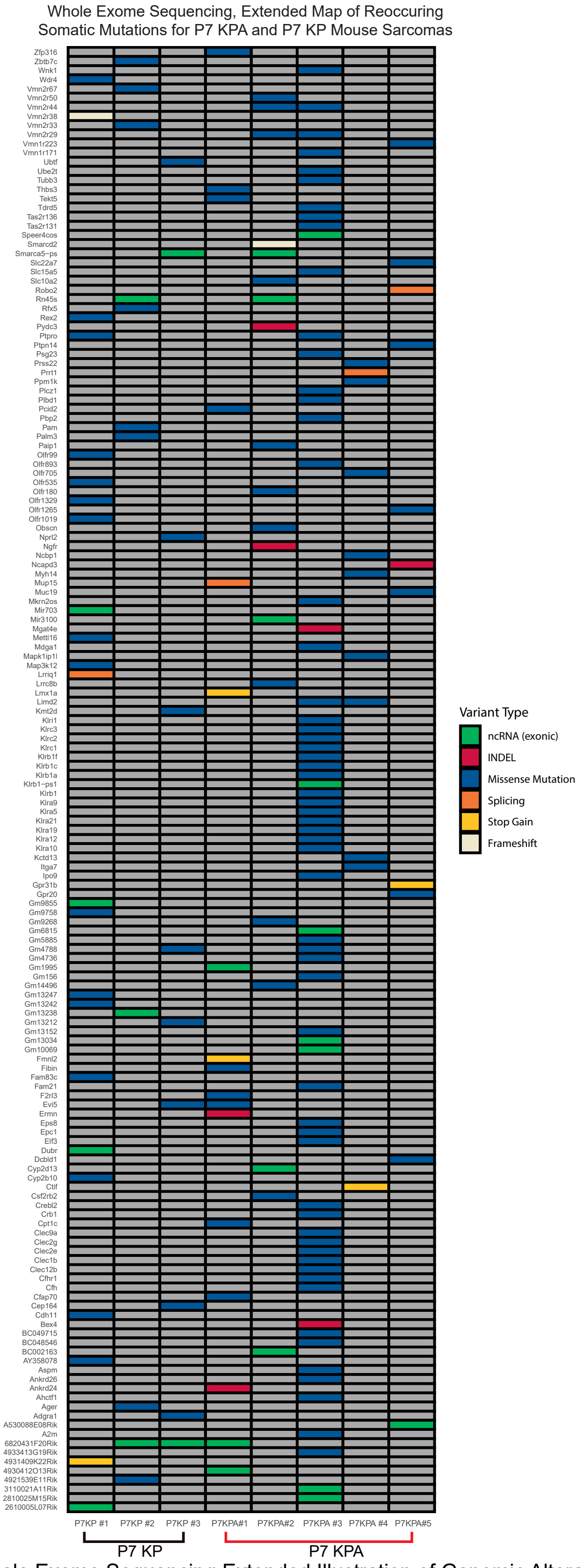

Supplementary Figure 10: Whole Exome Sequencing Extended Illustration of Genomic Alterations
Table showing most frequent recurrent mutations (each row is a different mutation) in sarcomas from P7 KPA and P7 KP mice (each column is a different mouse sarcoma). Mutations were identified via whole exome sequencing as described in the methods section. Specific mutation types are color coded as shown in the legend

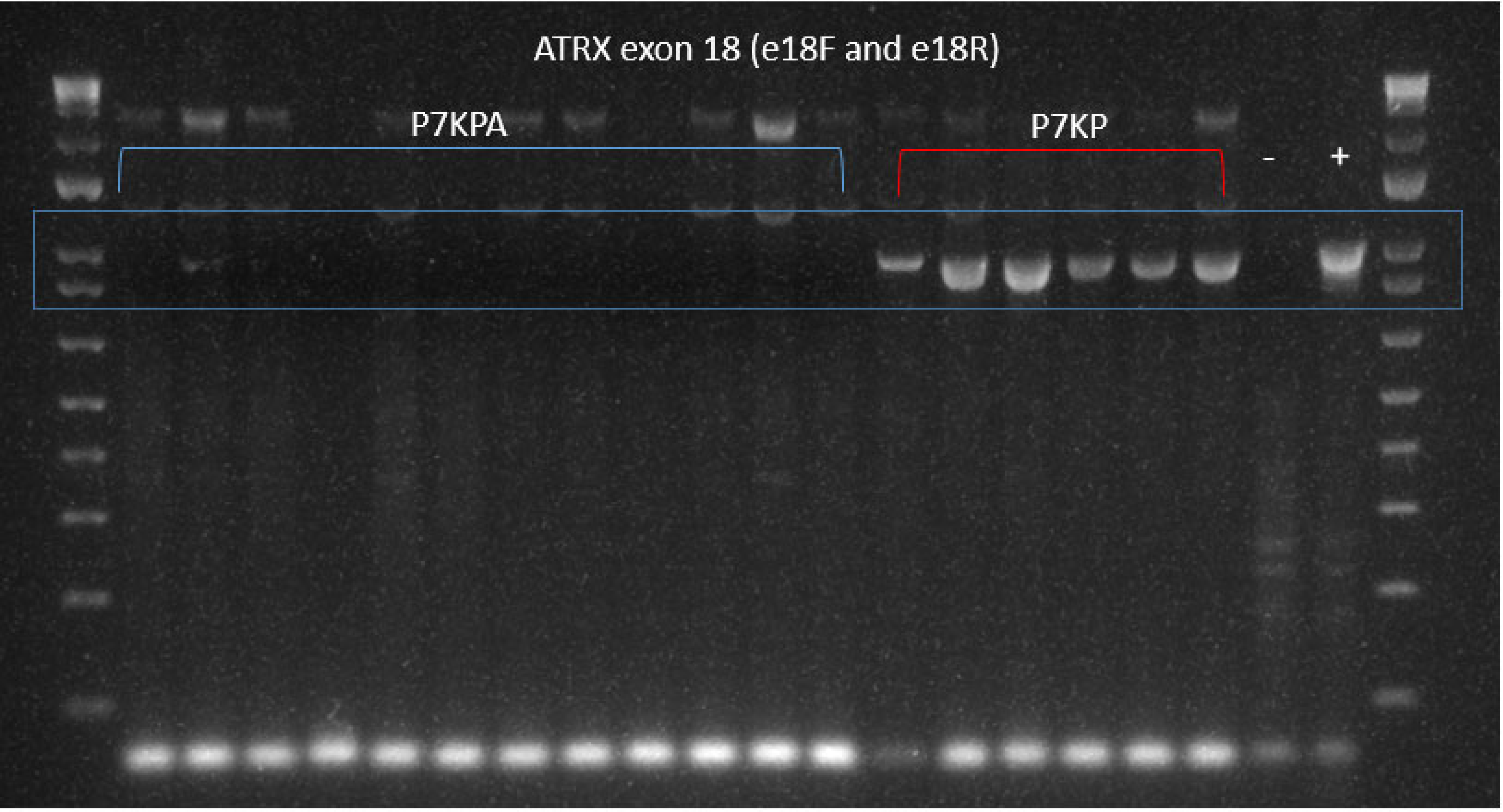
ATRX exon 18 (e18F and e18R)
P7KPA
P7KP
-
+

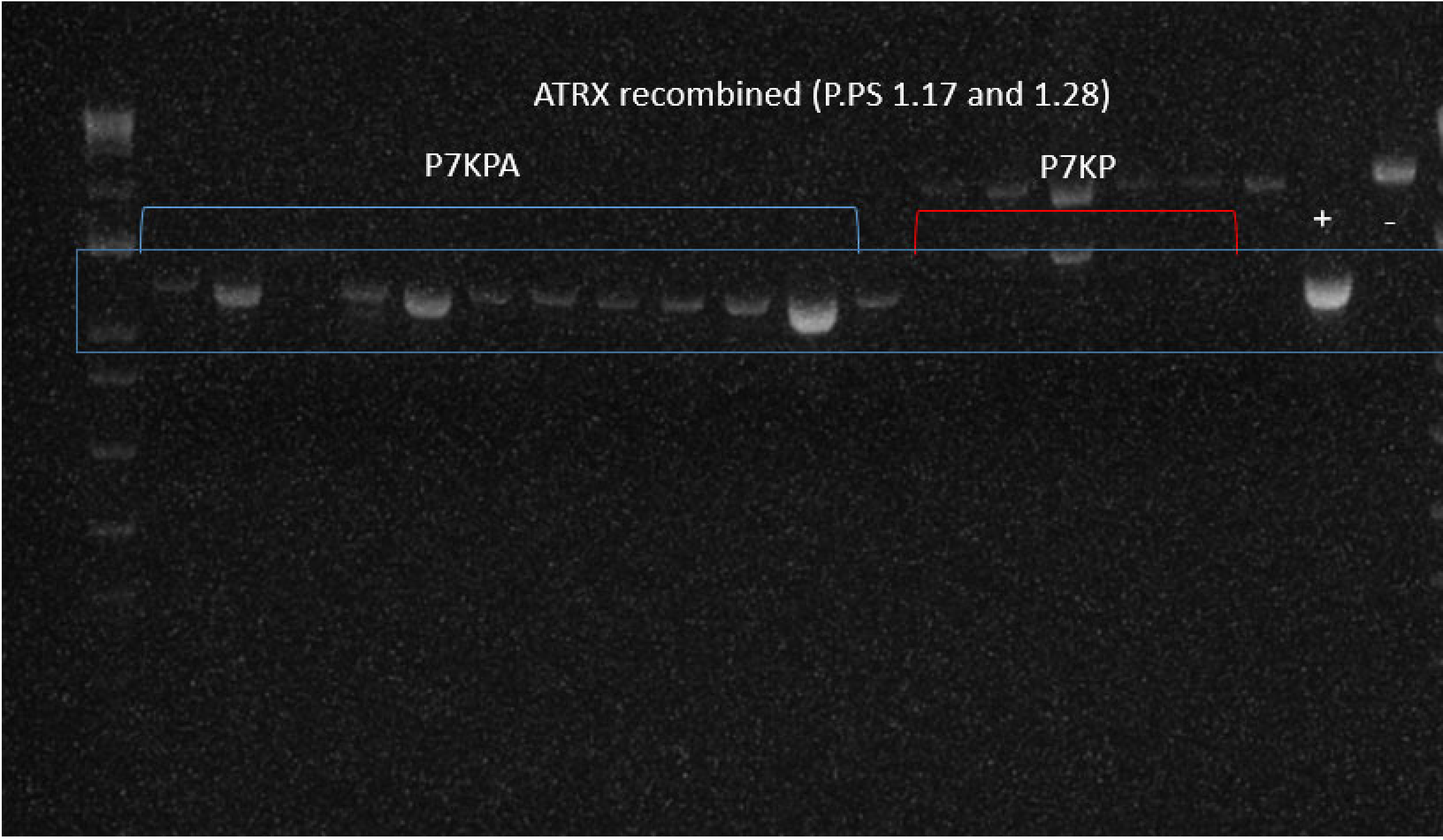
ATRX recombined (P.PS 1.17 and 1.28)
P7KPA
P7KP
+
-

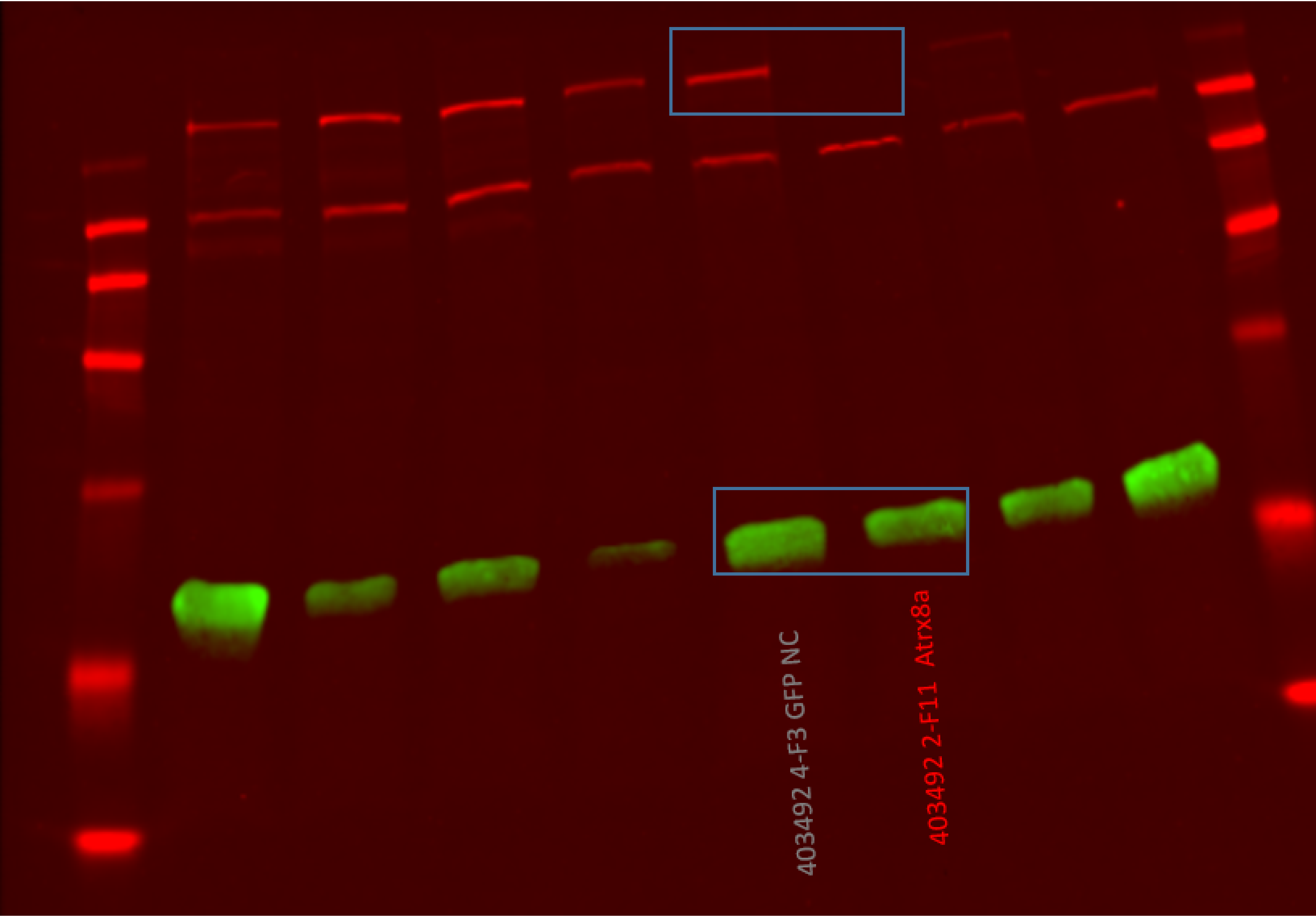
403492 4-F3 GFP NC
403492 2-F11 Atrx8a
